# Next Generation Sequencing-Based Molecular Marker Development: A Case Study in *Betula Alnoides*

**DOI:** 10.3390/molecules23112963

**Published:** 2018-11-13

**Authors:** Jing Tan, Jun-Jie Guo, Ming-Yu Yin, Huan Wang, Wen-Pan Dong, Jie Zeng, Shi-Liang Zhou

**Affiliations:** 1 Research Institute of Tropical Forestry, Chinese Academy of Forestry, Guangzhou 510520, China; planttj@126.com (J.T.); ymy920916@163.com (M.-Y.Y.); Whuan1819@163.com (H.W.); zengj69@caf.ac.cn (J.Z.); 2 State Key Laboratory of Systematic and Evolutionary Botany, Institute of Botany, Chinese Academy of Sciences, Beijing 100093, China; wpdong@ibcas.ac.cn (W.-P.D.); slzhou@ibcas.ac.cn (S.-L.Z.)

**Keywords:** *Betula alnoides*, next generation sequencing (NGS), simple sequence repeat (SSR), polymorphism, transferability

## Abstract

*Betula alnoides* is a fast-growing valuable indigenous tree species with multiple uses in the tropical and warm subtropical regions in South-East Asia and southern China. It has been proved to be tetraploid in most parts of its distribution in China. In the present study, next generation sequencing (NGS) technology was applied to develop numerous SSR markers for *B. alnoides*, and 64,376 contig sequences of 106,452 clean reads containing 164,357 candidate SSR loci were obtained. Among the derived SSR repeats, mono-nucleotide was the main type (77.05%), followed by di- (10.18%), tetra- (6.12%), tri- (3.56%), penta- (2.14%) and hexa-nucleotide (0.95%). The short nucleotide sequence repeats accounted for 90.79%. Among the 291 repeat motifs, AG/CT (46.33%) and AT/AT (44.15%) were the most common di-nucleotide repeats, while AAT/ATT (48.98%) was the most common tri-nucleotide repeats. A total of 2549 primer sets were designed from the identified putative SSR regions of which 900 were randomly selected for evaluation of amplification successfulness and detection of polymorphism if amplified successfully. Three hundred and ten polymorphic markers were obtained through testing with 24 individuals from *B. alnoides* natural forest in Jingxi County, Guangxi, China. The number of alleles (*N*_A_) of each marker ranged from 2 to 19 with a mean of 5.14. The observed (*H*_O_) and expected (*H*_E_) heterozygosities varied from 0.04 to 1.00 and 0.04 to 0.92 with their means being 0.64 and 0.57, respectively. Shannon-Wiener diversity index (*I*) ranged from 0.10 to 2.68 with a mean of 1.12. Cross-species transferability was further examined for 96 pairs of SSR primers randomly selected, and it was found that 48.96–84.38% of the primer pairs could successfully amplify each of six related *Betula* species. The obtained SSR markers can be used to study population genetics and molecular marker assisted breeding, particularly genome-wide association study of these species in the future.

## 1. Introduction

*Betula alnoides* Buch. Ham. ex D. Don (Betulaceae) is a fast-growing valuable indigenous tree species with multiple uses in the tropical and warm subtropical regions in South-East Asia and southern China. In China, its main natural distribution is in Yunnan, Guangxi, and Guizhou provinces [1]. The wood has beautiful texture, moderate density, low crack and deformation rate, and easy processing, making it suitable for floor timber and high-grade furniture making, high-class interior decoration and overlaid veneer [2]. The bark has anti-inflammatory [3], weight-loss and lipid-lowering properties [4,5]. In addition, *B. alnoides* forests have high ecological values in water conservation, maintenance of biodiversity and soil fertility, and carbon sequestration [6]. In recent decades, more than 150,000 hectares of plantations have been established in Yunnan, Guangxi, Guangdong, and Fujian provinces of China [7]. However, the majority of these plantations are established with seedlings of poor genetic quality, resulting in large variation for some traits such as diameter at breast height, tree height, stem form, wood density, insect resistance, and medicinal ingredients. Conventional quantitative breeding methods may take a long time to obtain elite varieties whereas molecular marker assisted breeding can greatly accelerate the process.

Molecular marker technology is an important tool for genomic studies, and the polymorphism of markers can reflect the same and discrepant features among individuals or populations at a certain level [8,9,10]. Simple sequence repeat (SSR) markers are widely used in population genetics and molecular marker assisted breeding, such as genome-wide association study (GWAS) due to codominant character, high polymorphism, wide distribution in the genome, neutral selection, abundant genetic information, easy detection, good reproducibility, and less demand for DNA templates [11,12,13,14,15]. However, the development of SSR markers is usually limited in species with little information on their genomic sequence. The advent of next generation sequencing (NGS) technology has overcome this restriction, and greatly promoted the genome sequencing of a species [16,17]. NGS has three advantages: (1) it can measure hundreds of thousands to millions of DNA sequences at a time; (2) NGS makes it possible to carry out a detailed analysis of the transcriptome and genome of a species, wherefore it is also known as deep sequencing; and (3) it is cost effectively compared with the first generation sequencing technology [18,19,20,21]. Therefore, NGS technology is increasingly being applied for the development of SSR markers.

*B. alnoides* has been proved to be tetraploid (2n = 4x = 56) in most parts of its distribution in China [22]. In some previous studies, allozyme, RAPD and AFLP markers were applied to assess the genetic diversity and population genetic structure of *B. alnoides* [23,24,25]. Guo et al. [26] developed 19 microsatellite markers of this species, and Sui et al. [27] adopted four SSR primers of *B. platyphylla* to study genetic diversity of *B. alnoides*. However, a small number of markers cannot meet the demand for molecular breeding like GWAS. At present, the analysis of the genetic basis of important traits has become a hot topic. GWAS overcomes some limitations of traditional gene mapping methods, and improves accuracy of gene-trait association and possibility of discovering new genes [28]. However, GWAS needs a large number of genetic markers, such as SSR [29] and SNP (Single Nucleotide Polymorphisms) [30]. Considering the limited information on *B. alnoides* genome as mentioned above, developing a large amount of SSR markers would be an efficient way to solve this problem.

In the present study, DNA-seq of *B. alnoides* was carried out to produce contig sequences containing SSR loci through NGS in order to: (1) analyze the distribution and characteristics of SSR loci in the genome; (2) develop a large number of polymorphic SSR markers; and (3) evaluate cross-species transferability of these SSR markers. The obtained SSR markers could be useful to promote further studies on population genetics and molecular marker assisted breeding of this and related species.

## 2. Results and Discussion

### 2.1. Information of Sequencing Data

**Figure 1:**
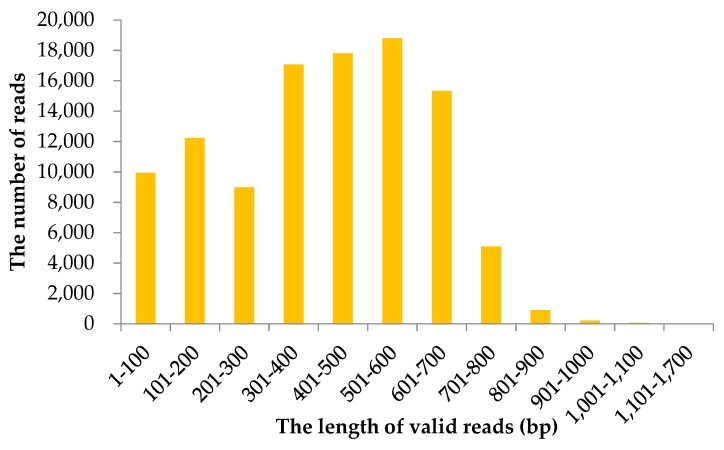
Length distribution of valid reads sequenced on GS FLX plus platform for *Betula alnoides*.

A total of 106,542 valid reads were generated using 1/6 of a run on the GS FLX plus platform. The raw sequencing dataset for *B. alnoides* was deposited into the NCBI SRA database (SRR7958615). The mean read length was 435 base pair (bp). Analysis on the length distribution of valid reads showed that 501–600 bp and 1101–1700 bp intervals had the maximum and minimum numbers of reads, respectively (Figure 1).

### 2.2. Distribution of SSR in the Genome

A total of 64,376 contig sequences, about 60.47%, of 106,452 clean reads, contained SSR loci. Among these, 25,431 sequences contained only one SSR locus, and the other 38,945 sequences contained at least two SSR loci. In total 164,357 candidate SSR loci were obtained (Table 1). The total size of examined sequences was 106,901,537 bp, and the size of the SSR repeat lengths ranged from 10 bp to 75 bp with a mean length of 16 bp. Mono-nucleotide repeats accounted for the largest percentage (77.05%) among all derived loci, followed by di-nucleotide (10.18%) and tetra-nucleotide (6.12%) repeats (Table 1). The frequency of tri-nucleotide, penta-nucleotide and hexa-nucleotide repeats was lower, being 3.56, 2.14 and 0.95%, respectively.

Our study found thousands of times more microsatellite sequences than those obtained by using the DNA library and the probe hybridization enrichment methods [31]. For example, only 58 microsatellite-containing fragments were obtained by screening genomic libraries with probes for *B. alnoides* [26] while 38 microsatellite sequences were obtained for *B. pendula* [32]. Among the abundant mono- to hexa-nucleotide repeats from the present study, mono-, di- and tetra-nucleotides were the major genomic microsatellite nucleotide types, while tri-, penta- and hexa-nucleotides were of relatively low proportion. Similar results have also been observed in other woody plants based on the same sequencing technology, such as *Prunus persica*, *Carica papaya* and *Citrus sinensis* [33].

### 2.3. Characteristics of SSR Sequences

It was found that 106,526 mono-nucleotide repeats (84.11% of the total) fell under 9–14 repeat number counts, and only four greater than 33 (Table 1). All the other nucleotide repeats were mainly within 3–8 repeat number category, 82.37% for di-, 96.91% for tri-, and nearly 100% for tetra-, penta- and hexa-nucleotide repeats, respectively.

**Figure 2:**
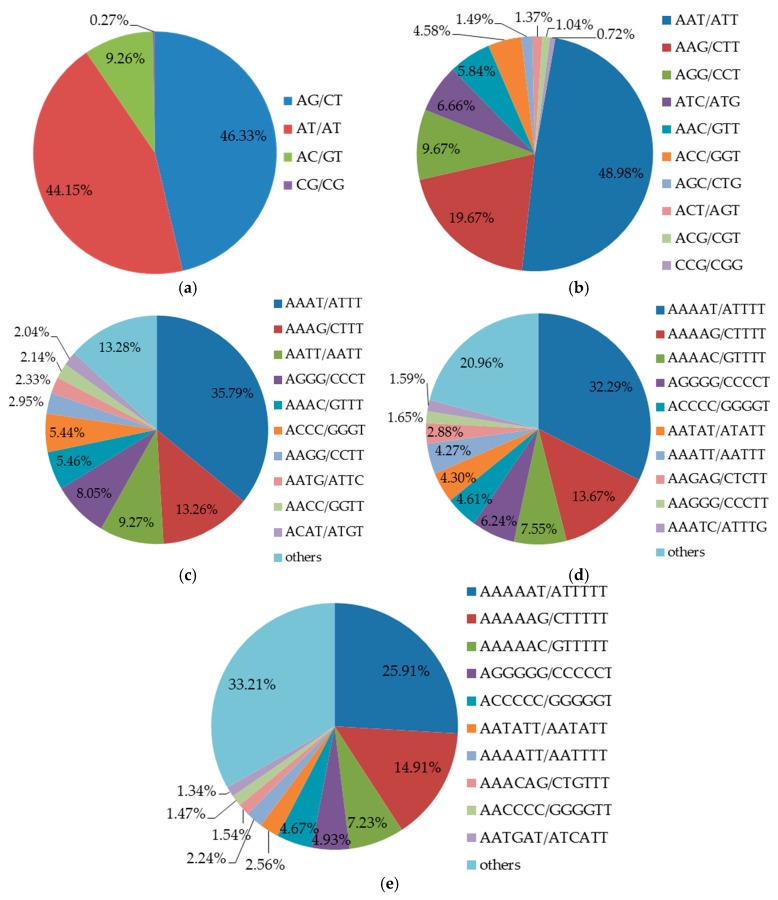
Percentages of different types of repeat motifs in the genome of *Betula alnoides*. (**a**–**e**) refer to di-, tri-, tetra-, penta- and hexa-nucleotide repeat motifs, respectively.

There were 291 types of repeat motifs in total. AG/CT (46.33%) and AT/AT (44.15%) were the most common repeat motifs in four types of di-nucleotide, whereas CG/CG accounted for only 0.27% (Figure 2a). These results are in accordance with previous studies reported by Gupta et al. [34] and Powell et al. [35]. In addition, the GC motif of lower frequency may be associated with cytosine methylation, which can be converted into a thymidine by deamination [36]. Among the ten types of repeat motifs in tri-nucleotide found in the present study (Figure 1b), the highest frequency repeat motif was AAT/ATT (48.98%), and the lowest was CCG/CGG (0.72%, Figure 2b). The main repeat motifs of tri-nucleotide were different from other plant species such as maize (CCG/GGC), rice (AGG/TCC) and barley (CCG/GGC) [37]. Repeat motifs AAAT/ATTT (60.85%), AAAAT/ATTTT (32.29%) and AAAAAT/ATTTTT (25.91%) observed in the present study were the most frequent in tetra-, penta- and hexa-nucleotide, respectively (Figure 2c–e).

### 2.4. Primer Screening and SSR Markers Polymorphism Detecting

**Figure 3:**
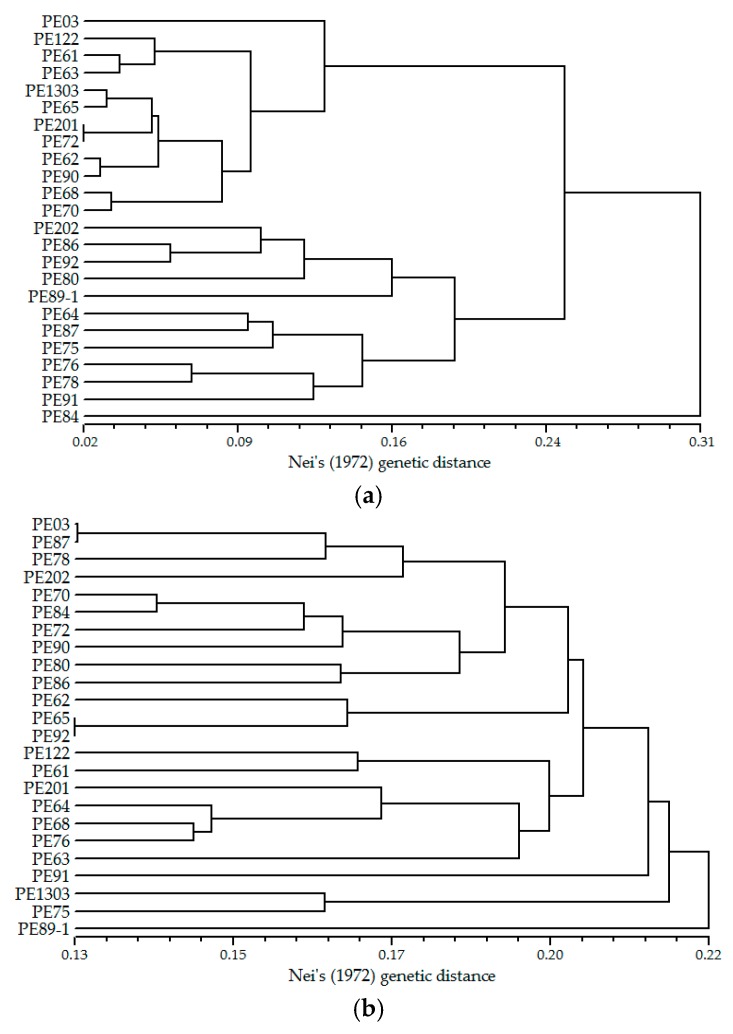
Phylogenetic relationship between 24 *Betula alnoides* individuals generated from UPGMA cluster analysis based on 19 (**a**) and 310 SSR markers (**b**).

In total 2549 pairs of SSR primers were designed from all candidate SSR loci, of which 138 pairs were excluded due to the occurrence of compound formation in repeat regions of relevant SSR sequences. The majority of nucleotide repeats were di-nucleotide (75.53%), followed by tri-nucleotide (20.24%). A total of 900 pairs of primers were randomly selected for evaluating the successfulness of amplification, among them di-, tri-, tetra-, penta- and hexa-nucleotide repeats accounted for 74.11%, 21.00%, 3.33%, 0.67%, and 0.89%, respectively, and 580 pairs of primers (64.44%) were amplified successfully with clear and distinguishable microsatellite bands. The optimum annealing temperature of these SSR primers was 60 °C as determined by a temperature trial with three individuals. Eventually, 310 primers were shown to detect polymorphisms in 24 samples from a natural *B. alnoides* forest in Jingxi County, Guangxi, China and are therefore designated polymorphic SSR loci. Characterizations of 310 polymorphic SSR loci are presented in Table S1, and the remaining 270 pairs generated monomorphic markers or fixed heterozygosity in 24 samples or had weak fluorescence signals in amplified products of most samples (Table S2). The fragment length of the amplified products was from 105 bp to 306 bp, which was within the expectation range (100–400 bp). None of the 310 SSR markers developed in the present study was the same as the 19 pairs developed in a previous study [26]. When we tested the usefulness of the 19 pairs developed by Guo et al. [26] for our 24 *B. alnoides* samples, it was found that all of them were identified as polymorphic loci (Table S3). Based on the previously reported SSR markers (Figure 3a) and the SSR markers developed in the present study (Figure 3b), a phylogenetic relationship was constructed between the 24 individuals of *B. alnoides*. It could be seen that the larger number of markers resulted in very different phylogenetic relationships between the 24 samples.

**Figure 4:**
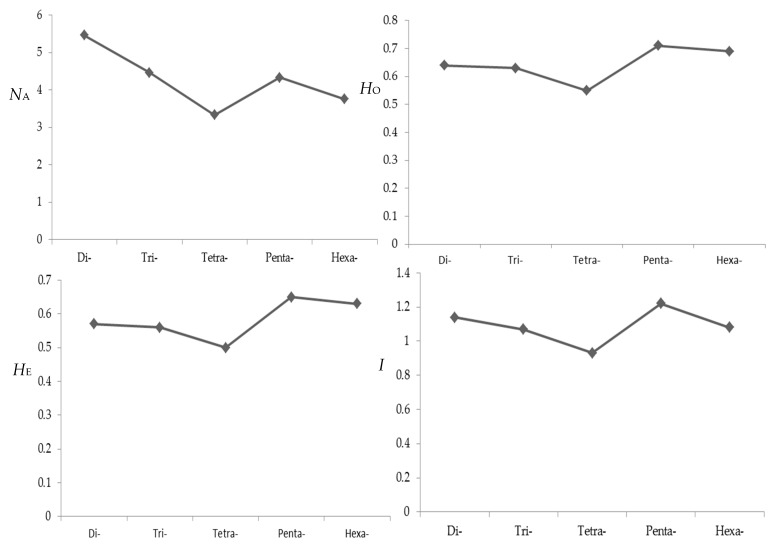
Influence of repeat type on polymorphism level of microsatellite markers. *N*_A_, number of alleles; *H*_O_, observed heterozygosity; *H*_E_, expected heterozygosity; *I*, Shannon-Wiener diversity index.

The genetic variation revealed by all polymorphic SSR loci for 24 individuals is also demonstrated in Table S1. The number of alleles (*N*_A_) of each locus ranged from 2 to 19 with a mean of 5.14, and 86.45% of loci had 2 to 8 alleles. The observed (*H*_O_) and expected (*H*_E_) heterozygosities of each locus varied from 0.04 to 1.00 (mean 0.64) and 0.04 to 0.92 (mean 0.57) respectively. The Shannon–Wiener diversity index (*I*) ranged from 0.10 to 2.68 with a mean of 1.12. The parameters of polymorphism decreased as nucleotide repeat varying from di- to tetra-nucleotides (Figure 4). However, this trend could not be seen for penta- and hexa-nucleotides due perhaps to their less occupancy. As a whole, the percentage of polymorphic loci with di-nucleotide repeats was larger than that with other nucleotide repeats. It was indicated that a selection of di-nucleotide repeats might be more effective to screen out polymorphic microsatellite markers, which is similar to the results of a previous study on *B. platyphylla* [38].

### 2.5. Cross-Species Transferability

Ninety six pairs of SSR primers were randomly selected to examine cross-species transferability among six related species in the genus *Betula*. The results showed that 48.96% of these SSR primers could be amplified in *B. platyphylla*, 73.96% in *B. austro-sinensis*, 82.29% in *B. cylindrostachya*, 78.13% in *B. fujianensis*, 79.17% in *B. hainanensis*, and 84.38% in *B. luminifera* (Table S4). These results thus indicated that these SSR primers had a high rate of transferability among the six species. High rate of transferability between species in the same genus has also been verified by Wang et al. [39] who discovered that 86.10% of SSR primer pairs developed from other *Prunus* species was transferable to *P*. *virginiana*. This is because the transferability of SSR primers across species is determined by the conservativeness of flanking sequences of microsatellite and their evolutionary stability [40]. The genomic differences between the related species are relatively small, making SSR primers highly transferable across specie. Thus, a large number of available primer pairs can be used in the genetic studies and molecular assistant breeding for the species in the genus *Betula*.

## 3. Materials and Methods

### 3.1. Plant Material and DNA Isolation

In total 24 individuals of *B. alnoides* were sampled from natural forest in Jingxi County, Guangxi, China. Among them, one sample was randomly selected for *de novo* genome sequencing, three samples were applied to screen out the successfully amplified SSR primers and optimum annealing temperatures, and all 24 individuals were used to evaluate polymorphism of the developed SSR loci. Six species in the genus *Betula* (Table 2) were used to assess cross-species transferability of these SSR loci. Fresh leaves were collected from each individual and dried separately with silica gel. Total genomic DNA was then extracted by modified CTAB method [41] and stored at −20 °C. The concentration and purity of DNA were determined by NanoDrop 2000 (Thermo Fisher Scientific Inc., Waltham, MA, USA).

### 3.2. Genome Sequencing, SSR Finding and Survey

*De novo* genome sequencing for *B. alnoides* was conducted using 1/6 of a run on the Roche 454 GS FLX + platform (454 Life Sciences, Roche Company, Branford, CT, USA) according to the process of Guo et al. [42]. For each sequence of raw data, the quality-control processes were conducted with the software Qiime (version 1.17, http://qiime.org/), which included: (1) the sequencing adapters were trimmed; (2) low-quality bases were trimmed with the index of the Q20 bases percent more than 90%; and (3) the ambiguous bases were deleted.

The MISA software was used to search SSR with following conditions: (1) the repeat number of the mono-nucleotide was at least ten; (2) the repeat number of the di-nucleotide was at least five; (3) the repeat number of the tri-nucleotide was at least four; and (4) the repeat numbers of the te-, penta-, and hexa-nucleotide were at least three. The characteristics of SSR were analyzed at the whole genome level.

### 3.3. SSR Primer Design and Screening

Primers were designed by Primer 3 with the following parameters: primer length = 18–30 bp; GC = 40–70%; temperature = 55–60 °C; product size range = 100–400 bp; and without secondary structure and dimer. SSR primers were synthesized by Sangon Biotech Co., Ltd., (Shanghai, China).

The optimum annealing temperature of SSR primers was screened out from 56 °C, 58 °C and 60 °C. The PCR reaction mixture (10 μL) contained 50 ng of DNA template, 150 μM dNTPs, 2.0 μM MgCl_2_, 0.5 μM forward and reverse primers, 1× PCR buffer (Tiangen Biotech Ltd., Beijing, China), and 0.04 U/μL of Taq DNA polymerase (Tiangen Biotech Ltd., Beijing, China). PCR was performed on the PCR system (Applied Biosystems Veriti) according to the following program: 4 min for initial denaturation at 94 °C; 31 cycles of 30 s for denaturation at 94 °C, 30 s at annealing temperature and 30 s at 72 °C; and 10 min at 72 °C. The amplified products were stored at 4 °C, and detected by 1% agarose gel electrophoresis. Successful amplification was judged by clear and distinguishable microsatellite bands.

### 3.4. SSR Markers Polymorphism Detection

Polymorphism of SSR loci was detected with the M13-tailed primers method [43]. SSR forward primers were synthesized and labeled with M13 sequence (5′-CACGACGTTGTAAAACGAC-3′) at 5′ end, and the reverse primers were not labeled. The M13 primer was labeled with a fluorescent dye FAM, NED, VIC, or ROX. The PCR was performed with a reaction mixture (10 μL) containing 50 ng of DNA template, 150 μM dNTPs, 2.0 μM MgCl_2_, 0.5 μM fluorescent forward M13-labeled primers and reverse primers, 1× PCR buffer (Tiangen Biotech Ltd., Beijing, China), and 0.04 U/μL of Taq DNA polymerase (Tiangen Biotech Ltd., Beijing, China). The PCR program was used as mentioned above.

The PCR products were detected using 3730 XL automatic sequencer (ABI Co., Foster, CA, USA). The genotyping of products was performed using GeneMarker V2.2.0 [44]. The number of alleles (*N*_A_) and observed heterozygosity (*H*_O_) were calculated with the results of genotyping in Excel 2010 (Microsoft Corporation, Redmond, WA, USA), the expected heterozygosity (*H*_E_) and Shannon–Wiener diversity index (*I*) were generated using ATETRA [45], which is specially applied for the analysis of tetraploid. The Nei’s genetic distance between the 24 samples was computed by NTSYS 2.1 [46] software. The software was also used to obtain clustering graph of 24 samples based on Nei’s genetic distance by the unweighted pair group method with arithmetic (UPGMA) method.

## Figures and Tables

**Table 1:** Distribution of the SSR loci in the genome of *Betula alnoides*.

Repeat Type	Repeat Number						Total	Frequency (%)
	3–8	9–14	15–20	21–26	27–32	≥33		
Mono-nucleotide	-	106,526	16,966	2328	821	4	126,645	77.05
Di-nucleotide	13,777	2601	296	37	15	-	16,726	10.18
Tri-nucleotide	5675	163	15	3	-	-	5856	3.56
Tetra-nucleotide	10,053	2	-	-	-	-	10,055	6.12
Penta-nucleotide	3509	1	2	-	-	-	3512	2.14
Hexa-nucleotide	1562	1	-	-	-	-	1563	0.95
Total	34,576	109,294	17,279	2368	836	4	164,357	100
Frequency (%)	21.04	66.50	10.51	1.44	0.51	0.24 × 10^−4^		

**Table 2:** List of six *Betula* species in China used for cross-species transferability of developed SSR markers.

Related Species	Collection Locality	Longitude (°E)	Latitude (°N)
*B. platyphylla*	Baihuashan Mountain, Beijing	115.57	39.85
*B. austro-sinensis*	Yangdongshan Shierdushui Provincial Nature Reserve, Lechang, Guangdong	113.39	25.28
*B. cylindrostachya*	Hill behind Cizhong Church, Weixi County, Yunnan	98.92	28.48
*B. fujianensis*	Luoboyan Natural Reserve, Fujian	117.57	27.43
*B. hainanensis*	Jianfengling Natural Reserve, Hainan	109.17	18.73
*B. luminifera*	Qinwang Mountain, Tianlin County, Guangxi	106.62	23.91

## Supplementary Materials

The supplementary materials are available online. Table S1: 310 polymorphic SSR markers validated in 24 samples of *Betula alnoides*. Table S2: The PCR results for 270 SSR primer pairs in 24 samples of *Betula alnoides*. Table S3: Validation of 19 SSR markers developed previously in 24 samples of *Betula alnoides*. Table S4: Amplification rate of 96 SSR markers for *Betula alnoides* in six related species of genus *Betula*.
