# Supplementary material for: Next Generation Sequencing-Based Molecular Marker Development: A Case Study in Betula Alnoides
Source: Molecules. 2018 Nov 13;23(11):2963. doi: 10.3390/molecules23112963 (PMC6278481; doi:10.3390/molecules23112963)
Supplement: Supplementary file 1 [file molecules-23-02963-s001.pdf]

Supplementary Materials

Table S1. 310 polymorphic SSR markers validated in 24 samples of *Betula alnoides*.

Primer Name	GenBank No.	Motif	Primer Sequence	Ta (℃)	N_A	H_O	H_E	I	Size of allels (bp)
BG0002	MH973665	(TA)8	F:TCATTCGATTGCAGCCATAACT	60	3	0.83	0.51	0.80	247-251
			R:TTTCGCCTTCTAAAACACCCCT						
BG0004	MH973666	(AT)8	F:TCTCCTGATTACATCTACCCGA	60	3	0.30	0.45	0.78	109-119
			R:TGCCAGAGTGTTAGAAAGGAGT						
BG0005	MH973667	(TC)8	F:TGTCTTCCACCTTTTCACCCAA	60	2	0.67	0.44	0.63	193-195
			R:TGGTGCATGAGTTGTAATTTCGA						
BG0006	MH973668	(TC)7	F:TAGACTGTCTACCTCGGCTCAA	60	4	0.88	0.74	1.36	105-111
			R:TGTTCAAAGAGTCCATCCCGAG						
BG0009	MH973669	(GA)9	F:ACCAACAGGAACCCATATCACC	60	11	0.71	0.71	1.74	216-260
			R:CCAGAGGATAGGATAGAGGCGA						
BG0011	MH973670	(TA)6	F:TGCTAGTACTCCAAGCATGAGG	60	2	0.86	0.53	0.83	277-281
			R:GGACAGTGGCAGAGCTTAAAGA						
BG0019	MH973671	(CT)7	F:ACCCAGCTATCTATCCCATGGA	60	3	0.62	0.61	1.02	126-130
			R:GTTGAGTCCACAATTCAGCACC						
BG0023	MH973672	(TC)7	F:AGCATTCACAGCAGATACCCAA	60	2	0.79	0.48	0.67	259-261
			R:TTTCTCCAGGGGATGAACAACC						
BG0025	MH973673	(ATA)7	F:AGTGTTACAACAGTCGCCTGAA	60	3	0.06	0.33	0.61	121-130
			R:AGCTTTATTCCACTTGGCTCCA						
BG0026	MH973674	(TG)6	F:GGAGTTCAGTTCTGGAGAAGGG	60	2	0.21	0.19	0.33	243-245
			R:TCATTTCAAATCCCCTCTGGCA						
BG0028	MH973675	(TA)6	F:TGTTCCTCTATTCATGTATGTGGA	60	3	0.17	0.57	0.92	128-132

			R:TCATTTGGGAACGAGTTTGTCA						
BG0029	MH973676	(TC)8	F:TACTCTTGACTGCAGGCTTGTT	60	5	0.54	0.49	1.02	247-255
			R:CCTCTTCCTCCTCTAGACCCAA						
BG0032	MH973680	(AG)11	F:AGAGTAGCGACGTGAATCATGG	60	10	0.92	0.81	1.83	219-237
			R:CATGCTAGCGTGACGAATTAGC						
BG0033	MH973681	(CT)11	F:GTTTTGGAACCTTCATGAGCCC	60	6	0.92	0.73	1.49	169-185
			R:GCCTTGTGCTTGGATTTTCCTT						
BG0036	MH973682	(AG)6	F:ATGGGGATTATGTGGTTATGCT	60	3	0.88	0.56	0.90	208-212
			R:AGAGTGCTTTGTCTGAATCCGT						
BG0037	MH973677	(AG)6	F:AGTGTGGAGTATCAGAGACCGA	60	4	1.00	0.66	1.15	105-111
			R:CCCCCTCTTCCTTTGCTTACTT						
BG0041	MH973678	(AT)7	F:AGGAAGAAGAACATGGGTGTGG	60	7	0.83	0.79	1.69	264-278
			R:TGAAAATTTACCCGCGATGGAC						
BG0046	MH973679	(AGA)5	F:CGAAGAAAATGACGGTGATCGG	60	3	0.79	0.55	0.91	192-198
			R:TGGTAAGCACTCACAAGCCTAG						
BG0047	MH978853	(AGA)5	F:AGACATTTCAAGCAAAAGGCCC	60	5	0.88	0.68	1.22	221-242
			R:ACAGCAACTATACTCCACAGCC						
BG0048	MH978852	(TTC)5	F:ACGAGTAGACTCCTAGAGATCATCA	60	4	0.63	0.58	1.14	194-209
			R:CGAGCGACACCAACTACCTAAT						
BG0049	MH978851	(AG)6	F:CCTTGTGAGAAACAACACCAAGG	60	4	0.92	0.35	0.68	141-147
			R:CTCATTCAGCCCTTCACCGAC						
BG0051	MH978850	(GA)6	F:TGTCTTCCTCGAGACTCAGACT	60	2	0.79	0.19	0.33	242-256
			R:GCATCTTTGGCATTTTCTGGGT						
BG0053	MH978849	(AT)6	F:TGTTGTCGGTTCGATAATCGGT	60	3	0.52	0.46	0.79	274-278
			R:CTGTGGAGTGTGGAGTATGACC						
BG0054	MH978848	(TA)6	F:TCACGCCTTCCATACAACAAAA	60	8	0.50	0.79	1.82	210-224

			R:GTCAGAGTAACAGGGTTGAGCA						
BG0055	MH978847	(CT)6	F:TCTATAGCTCGGTCGACCAAGA	60	2	0.08	0.08	0.17	151-153
			R:CTTCACCACTCAAGAACCCTCA						
BG0058	MH978846	(AATA)6	F:TACCCAGCAGGAGAGTTGGATA	60	3	0.50	0.56	0.88	244-252
			R:CCAGGTAGAGCTTTAAACCCGT						
BG0062	MH978845	(AT)6	F:CGTGGCATGACATGTCTGTTTT	60	5	0.71	0.74	1.43	268-280
			R:AGACAAGCGTAGACAAGGGAAA						
BG0064	MH978844	(TTA)6	F:GCTGAACCACCTTCATAGACCA	60	4	0.38	0.63	1.13	187-199
			R:AATACGACACGTGTCCAGTGAA						
BG0065	MH978843	(AG)6	F:TCCGGATTGTTCTAAGTCGGTG	60	2	0.63	0.43	0.62	136-138
			R:TCTCCTTCTTCTTCCTCTCCACT						
BG0069	MH978842	(AG)7	F:CCCATTTGCCAACGCTCATAAT	60	2	0.58	0.42	0.67	228-230
			R:CTGTGATGTAGGCGTACCCTTT						
BG0070	MH978841	(TC)12	F:CTCATACACGAACAAAGCGACC	60	6	0.54	0.62	1.27	209-219
			R:GTACGCCGAAAAAGAGGAGGTA						
BG0071	MH978840	(ATG)6	F:GTTGTCTTCTTGTGTGCCTGTC	60	7	1.00	0.82	1.79	206-224
			R:ACGACCTGCATCTACATCAACA						
BG0076	MH978839	(GA)6	F:GAAACCGACACACAGAGATGGA	60	3	0.17	0.16	0.34	101-105
			R:CCCGCATCTATCACTGCTACAT						
BG0078	MH978838	(GCT)5	F:CTTTTGCTGTGCAGTGGTTCTG	60	3	0.42	0.34	0.61	252-258
			R:CAACTTCATGGCAGCAATACCA						
BG0085	MH978837	(CT)6	F:GGCCTGCACTTGTTCCATATTC	60	2	0.04	0.04	0.10	240-242
			R:GACTGCCAAGAATTGCCCAATT						
BG0086	MH978836	(TC)12	F:CTCGTTTGGCTGTTTTCTGAGG	60	12	1.00	0.85	2.15	186-208
			R:TGAATGAAATCCAAGGGCGAGA						
BG0089	MH978835	(TA)9	F:TAGACTGTTGCTGCTTTTTCGC	60	2	0.13	0.12	0.27	250-254

			R:TAGTGGTATGACTGTGGGCAAG						
BG0091	MH978834	(CT)12	F:CTCTGTTGCTGTATATGCTGCC	60	17	1.00	0.90	2.50	140-180
			R:TCCATATCAAATCCCGGTCCAG						
BG0095	MH978833	(AG)15	F:TGTCTACTACATCCCCACAGTCT	60	11	0.88	0.88	2.25	222-250
			R:CGAGGATGCCATAATGAAGGGA						
BG0096	MH978832	(GCA)5	F:TTAATCCGGGCAGCCTTGAATT	60	5	0.92	0.68	1.26	132-147
			R:AGATTTGGACTCCGAAACTCCC						
BG0098	MH978831	(AC)6	F:TTAGCTCACCACTCACACAGTC	60	3	0.08	0.08	0.20	206-210
			R:AGCATGCAAAGCACGTGTTTAT						
BG0099	MH978830	(TA)6	F:AGAGACTCAAAAGTGGGGCAAT	60	10	0.79	0.84	2.04	229-247
			R:GGTGGAGTTGTTGACTTGTTCG						
BG0102	MH978829	(CTGCC)6	F:AAATAGCTGCCACCGTTCACTA	60	4	0.83	0.63	1.16	194-209
			R:CCCTTGTTTGTCAACCAACGAA						
BG0106	MH978828	(CT)9	F:TAGGCACACCCTTCTTTCGTAC	60	8	0.92	0.74	1.54	255-275
			R:GCTGGCTGTGAAGCAAGATTAC						
BG0118	MH978827	(GCTGT)5	F:AGCGCATAGGCCTTAACTACAA	60	6	0.92	0.74	1.48	244-269
			R:CAAACAACCTCACAGAACGCAT						
BG0119	MH978826	(ATGT)6	F:AGATTGATCGAGACCATGACGG	60	5	1.00	0.68	1.25	241-257
			R:ATAGGCACCAACTCAGGACATC						
BG0121	MH978825	(AG)7	F:CCAAACTAAGAAAGTCCCGAGC	60	6	0.38	0.71	1.38	220-230
			R:TCTCTGGGTTCGTAGGCTTAGA						
BG0122	MH978824	(TA)8	F:ACTTGAGTCGAAACCATCTAACCA	60	7	0.58	0.74	1.57	250-262
			R:GACCATTGCTCCCGTTAAATCG						
BG0124	MH978823	(AT)11	F:GCCCAAAATATGACGCTCGATC	60	9	0.88	0.80	1.85	101-121
			R:GGTGCTCGAGCGTCAAGNCT						
BG0126	MH978822	(GAA)9	F:ACTGTAAGGGGGTAAGGACTCA	60	3	0.54	0.50	0.87	255-264

			R:TTATTCAAGACAGCTGGAGCCC						
BG0127	MH978821	(AT)8	F:GACTCGTGTTGCGCCATTTAAT	60	7	0.67	0.82	1.85	165-177
			R:GGGAAGCACTAACACTCTCCAA						
BG0128	MH978820	(GA)6	F:CCGAGAGAGAGAGGGAGATACC	60	13	0.92	0.78	1.89	224-286
			R:GTTTGGGTCATAAACGGGTCAC						
BG0129	MH978819	(CT)8	F:GAGAAGTCAGAACCCTTCGCTT	60	8	0.92	0.74	1.60	158-178
			R:GCAATTGATAGCCTCCTCCGTA						
BG0132	MH978818	(GA)10	F:TGATTCATCGTCAGCCACAAGA	60	3	0.13	0.13	0.28	204-212
			R:AGCACACGTACGTACTAGTGTG						
BG0141	MH978817	(TG)8	F:AACTCCCACCCCTTACCTTAGT	60	3	0.38	0.40	0.72	278-282
			R:TTCCAACTTTCCTTCCCCTTCC						
BG0145	MH978816	(AT)6	F:ACTCCTGTAGGGCCTAGGAATT	60	4	0.96	0.63	1.08	142-148
			R:GAGGAGAATGGAGAGGGAGAGT						
BG0148	MH978815	(TC)9	F:GTGCCAAAGTGTATTCGGCTTT	60	19	0.96	0.92	2.68	179-217
			R:GCGAAGGCGTACAATGTTTGTT						
BG0149	MH978814	(AT)6	F:AATAGGGACGGAACCAGTGTTG	60	3	0.67	0.66	1.08	138-146
			R:CCATGGCTCCCCCTTGATTTTT						
BG0157	MH978813	(AAT)7	F:GGCATTGTCGCTAATTGCACTT	60	5	0.75	0.71	1.35	224-236
			R:TCAAAGTTGAGCTCACCTGGTT						
BG0158	MH978812	(TA)6	F:CGTTTGTGCGCAGATATGGTAG	60	3	1.00	0.51	0.75	221-225
			R:ATTTGCAGAACCCTGGCTCTAA						
BG0159	MH978811	(TA)13	F:CGTCTAATCCATCCATGCATGC	60	8	0.78	0.75	1.63	145-159
			R:AGACAGATCCTAGAGCTCTGCA						
BG0162	MH978810	(AT)6	F:ACGTTTAGCAGATGTTGTACTTGC	60	4	1.00	0.66	1.17	258-268
			R:TCCAATAGCAAATCAAATACCCAAA						
BG0163	MH978809	(CT)8	F:TAAAGTTGTCCCGTCCTTGCAT	60	8	0.75	0.82	1.87	237-253

			R:AGATGCATGACGAGAAGAGCAA						
BG0164	MH978808	(TC)12	F:TTTCAATTTCTCTGCGACCCCT	60	8	0.67	0.70	1.50	259-279
			R:GCCGCAATAGTAATCCAATCCG						
BG0165	MH978807	(AG)12	F:CTCTAGGGTTGCGCATTGAAAC	60	8	0.75	0.65	1.42	212-230
			R:GCAGCTGCCTTTACAAGACAAA						
BG0167	MH978806	(AT)6	F:GGCTTAGAAGATCCATGGCGTA	60	3	1.00	0.64	1.05	165-169
			R:GCGCTTCAGGTATATCCATCCT						
BG0169	MH978805	(AT)8	F:GCGCCCTTCTGCAATTTCTAAT	60	4	0.46	0.40	0.76	154-162
			R:TGCTGAAGGAAATCCAGTGAGG						
BG0174	MH978804	(GA)6	F:GAGATAGTGCTTGGAGAGACCG	60	5	0.63	0.75	1.45	241-255
			R:ACACAACACAACACAAGTCACG						
BG0175	MH978803	(ATAA)5	F:CGCTCATGTAAGCACTCCAATG	60	4	0.88	0.71	1.48	205-217
			R:ACTCCACTTTAACTTGCGTTGC						
BG0176	MH978802	(GA)7	F:CAAGAGAGATGGAGATCGGCTG	60	5	0.46	0.60	1.19	157-179
			R:CTCTCAATCTCTCTCTCACGCC						
BG0179	MH978801	(TTC)7	F:TTCAAAGGTCACCGATACGTGT	60	4	0.50	0.64	1.10	183-195
			R:GAATGACTGAGAATGGCAGCAC						
BG0184	MH978800	(TTTC)5	F:ATATTGGCGCTTGATCCCTAGG	60	4	0.83	0.68	1.24	266-278
			R:AACTCTCTCGCTCTCTGTCTTG						
BG0188	MH978799	(ATG)6	F:GACAAGTCCCCAGCATTACAGT	60	6	0.79	0.69	1.37	121-139
			R:GCTTCATCACTCCTTTCGAGGA						
BG0189	MH978798	(AAT)7	F:AGTGGTTCATATATGGTGTCCACA	60	4	0.67	0.52	0.88	153-165
			R:CAGCCCACTCTAATACCGTGAA						
BG0193	MH978797	(TC)6	F:TATTCCCTCCGCTCTCTCTCTC	60	4	0.54	0.43	0.81	180-188
			R:TGCATTCCTCCTTGTTCCAGAA						
BG0201	MH978796	(TG)6	F:AAGTGACTTTTCGTGTCAACCA	60	3	0.25	0.60	0.97	259-263

			R:ACCGGTACACAGCTTCTACAAG						
BG0205	MH978795	(AAT)7	F:TTTTGCTGAGCCCATTTTCACT	60	9	0.96	0.78	1.84	154-181
			R:ATCCATGGAAACTGGCTGTTCT						
BG0207	MH978794	(GA)7	F:AGAAAGAGATGAGCGTTTGGGG	60	5	0.96	0.61	1.11	101-109
			R:CACCATCTCTCTCTCATAACGCA						
BG0212	MH978793	(TA)9	F:AGGACATGCATGATGAAGTGGT	60	3	0.63	0.54	0.83	110-114
			R:GCCAACCCTTGATGTAAGCTTG						
BG0216	MH978792	(ACC)5	F:TCTGACAAACACGTCTGGATCC	60	5	0.92	0.72	1.44	258-276
			R:CCCTCGATCGATGTCTCCATTT						
BG0220	MH978791	(AATA)5	F:CCTATGGGCTGACACTGAATGA	60	2	0.29	0.25	0.41	164-168
			R:AACAAATGCAATTTCAGGGCAC						
BG0221	MH978790	(AG)6	F:TGAAGATGATGCTAGCGTCACT	60	3	0.08	0.08	0.20	268-272
			R:TGCCCCTAGTATTGGTCGACTA						
BG0222	MH978789	(AT)7	F:ATCGATCGAGACCTAGCTAGCT	60	4	0.50	0.42	0.79	156-162
			R:AGGCCGTGCACTGAATATTAGT						
BG0226	MH978788	(AG)10	F:CTCTCCCTTTCAAGTAGGTCGG	60	6	0.38	0.42	0.90	252-262
			R:CTCCATTTTGCTGTGGTTGGAG						
BG0232	MH978787	(TC)6	F:TGCACCTGAAGAGTTTCCAAGT	60	3	0.71	0.47	0.71	242-248
			R:ACCAAAGAGTTGTGACCATAGCA						
BG0233	MH978786	(TC)7	F:AAATAACGTCGTGTGGCACTTG	60	3	0.75	0.49	0.77	231-241
			R:TTCTCTCTACAATGGCGGAGAG						
BG0236	MH978785	(AT)7	F:GGATCAACGACATCTTCCTCGA	60	4	0.50	0.54	0.98	158-166
			R:CGCAGATTACTTGATTCAACAAGA						
BG0238	MH978784	(AG)9	F:CTACAACGCTCGTCCTCTTCTT	60	8	0.46	0.80	1.77	236-250
			R:CGCTGGAAGAGGGGCTAATATT						
BG0239	MH978783	(CA)7	F:TACCTCTTCAAGCCAACACACA	60	4	0.17	0.80	1.77	146-152

			R:CCCTTAACTAGACGTCTCAGCC						
BG0240	MH978782	(TA)8	F:TTTTTCGACGAGTTTTTGGCCC	60	3	1.00	0.65	1.08	275-279
			R:TCTTGTAACACTCCCCTCAAGC						
BG0245	MH978781	(TC)8	F:GAAACAAACGGGCCTTAGTGAA	60	6	0.71	0.81	1.75	206-216
			R:ACCGGATGTACATATGCAGGAC						
BG0248	MH978780	(CT)6	F:GAACAGACACGTCGTTTGCATG	60	3	0.75	0.58	0.30	264-268
			R:GGATCCCGTCATGTTCCAACA						
BG0251	MH978779	(AAT)5	F:AGCGTAAAGTGCTTTCAAATACA	60	3	0.04	0.08	0.20	214-220
			R:TGCCAGTAGAAGTAATCGTGCA						
BG0252	MH978778	(GA)12	F:ACCTTTGGATTTGAGCAAAGACA	60	15	0.96	0.87	2.27	161-199
			R:ACTGTTAGCAATGGTCAGTGGA						
BG0260	MH978777	(TTC)6	F:CACAAGTTACACAACTCACGCA	60	4	0.25	0.26	0.57	142-154
			R:CGAAGAAGAACAGTCTGACGGA						
BG0262	MH978776	(AT)6	F:GTTGTGGCAACCCGATTTCATA	60	2	0.08	0.28	0.45	167-169
			R:TTGGTTATGGTTCCCGATCAGG						
BG0267	MH978775	(TC)9	F:AGAGTACGGTTCGTGTGTAACC	60	4	0.50	0.42	0.80	202-218
			R:CTTTTCCTGCTGCTGCTTCTTT						
BG0275	MH978774	(AC)8	F:ACGTATGCAGCTATCCAATGAA	60	3	0.08	0.08	0.20	276-280
			R:TGTGTTAGGGTCCGAATCTAGT						
BG0285	MH978773	(TA)10	F:GACCCAAAAGCTCAGCAGTTTT	60	3	0.04	0.26	0.51	225-235
			R:AGGAAATCGCCCTTGACTTATCA						
BG0293	MH978772	(TAAT)5	F:TTGAGCATGTCAAATAGCATTTCT	60	2	0.13	0.12	0.23	212-216
			R:TAAAAGTACGTGCAGCTAGGCA						
BG0295	MH978771	(AG)9	F:TTTGCACCGGATAAAAGCACTG	60	7	0.54	0.78	1.13	105-121
			R:TATCAACGACCCTCCTCTGTCT						
BG0296	MH978770	(AT)18	F:CCAAGAGCCATCCTCACTCTTT	60	5	0.88	0.71	1.37	242-250

			R:GTCATTGCCTCCACTCCACTAA						
BG0299	MH978769	(GA)7	F:CAACGCAATCTCAAACAGGTCC	60	4	0.54	0.62	1.06	272-278
			R:TCCTACTCTCTAGCGTGGTTCA						
BG0301	MH978768	(AT)6	F:TGGAAAAGGATCCAGAGAAGCC	60	7	0.75	0.65	1.40	247-259
			R:GTATGGAGGGAAAGCTTTGCAC						
BG0302	MH978767	(AAT)9	F:GCAGCCACCAAAGAATACACAG	60	3	0.54	0.47	0.82	215-224
			R:CAACCACGCTTCTTTACTGCAA						
BG0304	MH978766	(TA)6	F:CCCTTCTCCCTTTCCCTCTTTC	60	3	0.21	0.50	0.86	101-113
			R:ACCCCATTAATCACCTTCCCAC						
BG0306	MH978765	(AG)9	F:AATCCTTGGAGAACGAGTTGGG	60	7	0.88	0.74	1.53	180-200
			R:GACATTCTCGTCTCGGGTAACA						
BG0307	MH978764	(AAG)5	F:CGGTTTGGAGAAACTTGCAGAG	60	3	1.00	0.66	1.08	236-254
			R:GCCAAAGCCAAACTCAATCTCA						
BG0314	MH978763	(AAT)6	F:TGAACTATCCTCATGGCCTTGG	60	3	0.29	0.33	0.61	132-144
			R:TGAATATTTGGCCACCCCTTCA						
BG0315	MH978762	(GA)10	F:TGCACGTCATTTTTAGCAAACA	60	2	0.09	0.09	0.18	137-145
			R:TTGAGCAGAGGGTTCAAGGATC						
BG0320	MH978761	(TC)6	F:CTCGAACCCCATCTTCTCCTTC	60	9	0.75	0.67	1.42	225-243
			R:AAAGACAGTGTAGTCCGTGCAT						
BG0324	MH978760	(TA)6	F:AATTGGGTGTTTGACGTTCACG	60	10	0.50	0.63	1.47	102-128
			R:AGCAATAGGAGGGGCTTAGAAA						
BG0325	MH978759	(TC)7	F:GGTGTACAGTGTCATGTCCCAT	60	4	0.88	0.72	1.33	117-125
			R:GGAAAGGGAAGGGAAGAGGAAG						
BG0328	MH978758	(TA)10	F:TTGTGTGTCCGAATGAGGTGAT	60	5	0.67	0.47	0.78	170-180
			R:CGACGACGAGAAAAGCAAGATG						
BG0329	MH978757	(AT)6	F:AAAGTGGGGTTGGAATGATCGA	60	4	0.35	0.64	1.13	233-241

			R:GCCAAACATGCCCTTTCCTTTT						
BG0331	MH978756	(CT)6	F:TTTCCCCACTCTCATCCCAAAG	60	4	0.92	0.73	1.35	137-145
			R:GAGGATAGAAAGAGGGCAGAGG						
BG0334	MH978755	(TTC)5	F:CGTCATCACTGATAAGGCCACT	60	2	0.08	0.08	0.17	223-226
			R:TCGGGTTCTCGAAAAGTTACCT						
BG0335	MH978754	(TTG)6	F:TGGTTGGAGGATTGAGACACAG	60	4	0.54	0.47	0.94	259-268
			R:CCACAGCACAACAACAACAAGA						
BG0337	MH978753	(CT)7	F:TGTGTACTTGCAATGCAGCATC	60	3	0.25	0.23	0.46	268-272
			R:AGAAAGAAGGGAACAGCGCTAA						
BG0338	MH978752	(AAAT)6	F:CCTCACTTGGCAGATGCAAAAA	60	6	0.75	0.80	1.67	219-239
			R:GGGACATTACTAAATCCCAGAAGC						
BG0339	MH978751	(GA)6	F:GGAGGATCTAACGGCAGAGAAG	60	2	0.25	0.23	0.38	181-183
			R:ACTCCTCAGCTCAACTCTCTCT						
BG0340	MH978750	(GA)7	F:GTGGAAGCCCATCAAAAGTTCC	60	13	0.88	0.74	1.75	222-250
			R:CGCAAAGGCACCTTTATTGTGA						
BG0342	MH978749	(TA)6	F:CTTCATGCAAATGCCACACTCA	60	2	0.08	0.08	0.17	237-241
			R:CCCACATGTCGCAAATCTAAGC						
BG0345	MH978748	(AT)10	F:ACCCTTAAAAGCTTGAGGTTCAGA	60	4	0.21	0.22	0.52	158-166
			R:CAGTGGAGGTTTAGGTTTCCGT						
BG0346	MH978747	(AG)6	F:CTTTGACTCCCCTCTCCACTTG	60	5	0.38	0.37	0.79	135-157
			R:CCAGCTAGCCTCTTGATCTTCA						
BG0347	MH978746	(AAAAT)5	F:TGATAAATGGGAATCAGCGGGA	60	3	0.38	0.59	1.02	130-140
			R:AACAACTCTTGCTTGTCAACGC						
BG0349	MH978745	(AAT)5	F:CATGCCTTTGAGGGGTTCATTG	60	2	0.28	0.50	0.69	155-161
			R:TAGGGCTAGTTCCTTACACCCA						
BG0350	MH978744	(ATT)6	F:TCGGAAATGCAAATTGAGCTGA	60	4	0.50	0.68	1.21	196-205

			R:TAATCTTAAACAACCGCAACCG						
BG0354	MH978743	(AG)10	F:TTAACCAAACAGTCCAGGGGAG	60	7	0.58	0.66	1.32	158-179
			R:ACGAGCAAAATGTAACCGTGAA						
BG0356	MH978742	(AT)8	F:TTGGGCTGCATACTCCTCAATT	60	8	0.50	0.73	1.63	111-129
			R:GCACTCTCTAATGCCAAACTCG						
BG0362	MH978741	(TTA)12	F:ATTGAAATCTGTTGACGCCGTC	60	11	1.00	0.83	2.00	206-236
			R:TGTTGCGCTTTCTCTTGCATTT						
BG0367	MH978740	(AG)11	F:AGTAACCAACCTTGCTACCACT	60	9	0.96	0.83	1.93	100-122
			R:TTGCTACCCAACAAAGCACAAG						
BG0369	MH978739	(TC)10	F:CATGCAGAACCCTCTCTTCCTT	60	8	0.88	0.75	1.62	219-239
			R:CCCCTCACGTTAAACAAGCAAG						
BG0370	MH978738	(TC)8	F:ACACAATCCTCCAAGGTTCATCA	60	11	0.88	0.77	1.77	141-169
			R:TGCATGTTTTCGCCATATTGGG						
BG0374	MH978737	(TC)7	F:ATCACACAGGCTAGGGTTTCTG	60	4	0.88	0.65	1.11	130-136
			R:GAAAGATGACGCTCGTGGGT						
BG0376	MH978736	(AG)6	F:CCCCCTTCTTCTCCCTTTCTTC	60	2	0.13	0.12	0.23	131-135
			R:CCGAAACTTCCCTTCTTAGGCT						
BG0377	MH978735	(AC)24	F:GGCCATCCCTTCACCTTATCTT	60	13	0.42	0.46	1.22	216-250
			R:ACACCCAGGTAAGCACAAATCA						
BG0379	MH978734	(GA)8	F:AACTTTTCTTCCATGCTCGCTG	60	17	0.83	0.92	2.62	129-177
			R:TTCAACAGAGATACCTCGGCAC						
BG0380	MH978733	(TA)8	F:AACGGAGAAAATATTGACCATGTT	60	4	0.54	0.72	1.32	112-116
			R:TGGTGTGGTTTATATGATGCCCA						
BG0386	MH978732	(TC)6	F:CCTCAAATGTTAGAGCTTTACGCC	60	6	0.17	0.45	0.88	100-118
			R:CGACATCCATTTTGGCTCGC						
BG0392	MH978731	(AAT)5	F:AAATCCCATCATCCACCGTTGA	60	3	0.42	0.37	0.66	207-213

			R:ATGTCAACGTAAGCACCCAGAT						
BG0395	MH978730	(TTA)11	F:AGACTGGAATTTAACATCGACCCA	60	5	0.50	0.76	1.51	272-284
			R:TGGGGATAGATATTCGATCTTTTCG						
BG0396	MH978729	(TGA)6	F:GGCAATTATGAAGTCATGGCTGA	60	2	0.88	0.50	0.69	201-204
			R:GCTTTTGCAAATGCCTAATGCT						
BG0397	MH978728	(AATGGA)5	F:ACTAAATTTAAAGGGGCCGGGT	60	3	0.79	0.57	0.93	166-178
			R:ACCAAATTTATACGGAGCCGGT						
BG0400	MH978727	(AT)6	F:CTCGAGGTTCTCAAACTGTGGA	60	4	0.71	0.59	1.05	246-254
			R:CCCCCACTTAAGGAAGAGGTTC						
BG0402	MH978726	(AT)7	F:TCCAGGAGTGCTCTTAAGGAGA	60	7	0.54	0.75	1.55	231-251
			R:GCTTGGTATCACTCCCTCAACA						
BG0407	MH978725	(CT)6	F:AAGGCTTCCGTTCTCTCATCTC	60	3	0.88	0.61	1.01	123-131
			R:AGAGAGATGGAGTCTGCTCAGT						
BG0411	MH978724	(AAAT)5	F:TGCTTGGGCCTAAAGAGGTTTA	60	2	0.58	0.41	0.60	121-125
			R:TGTGTAATTGCCAAGAGCTTGC						
BG0412	MH978723	(ATT)5	F:TCGTGAACAACTTGTGAATTGCA	60	3	0.50	0.38	0.63	262-268
			R:CGTGACAAAAAGGTGGAGCTTT						
BG0413	MH978722	(ATA)6	F:CCCACCCTTGTCCTGGTATTTT	60	2	0.38	0.30	0.48	216-219
			R:CACCCTTCCAATCTGAGGGAAT						
BG0414	MH978721	(TA)7	F:GATAGCTAGGTAGGCCAGCATG	60	4	0.83	0.67	1.21	156-170
			R:TAGGTGGTGAATATGCTGCCAA						
BG0415	MH978720	(AAT)10	F:ATCATGAGGTTGCCTGGGATTT	60	10	0.85	0.82	1.94	190-226
			R:TCCTTGGAACGGACTTTTCACA						
BG0416	MH978719	(TG)6	F:TCCAGCTCTCTGTCTCTCTCTC	60	4	0.83	0.67	1.21	220-230
			R:TCGACTGCCTTGGGTAAAACTT						
BG0419	MH978718	(TA)8	F:GTCACTTGAATCACGATGCCAA	60	7	0.88	0.73	1.51	217-231

			R:AGCACCTTAGTGGAACCATGAG						
BG0422	MH978717	(TA)8	F:GGTCACACGTTAAAGCCACTTG	60	4	0.79	0.61	1.06	224-236
			R:TACATGCTGCCGCCTTGTATAT						
BG0426	MH978716	(GAT)5	AGAGAGAGACAAGAAAGGGGGA	60	7	0.87	0.67	1.36	169-202
			R:CAAACCCACGCAGATTCACAAT						
BG0427	MH978715	(AG)12	F:CTGAGGCATGTTTGGTTTGGTT	60	10	0.83	0.82	1.92	141-161
			R:TATGAGACACGGGACTGTGAGA						
BG0432	MH978714	(TAAA)5	F:ACAAGAGCTATTGAAGAAAACACA	60	2	0.04	0.19	0.33	264-268
			R:GCACGTGCTAAAAGAATCTGCA						
BG0434	MH978713	(AAATAA)7	F:AACCATTGTTCACCAACGATGT	60	6	0.48	0.67	1.30	110-140
			R:AGTCCTGAATTGATAGGTGTGTGA						
BG0437	MH978712	(CTT)5	F:CGCCTAGCACTAATCCATACGT	60	3	0.08	0.23	0.45	249-261
			R:AGGCTACTTCAGTGTTGCTTGA						
BG0438	MH978711	(CGC)5	F:AGACTGAACCTGCAAAAATCGC	60	2	0.50	0.37	0.56	239-242
			R:CCCCCATCATCAACAACTCCA						
BG0441	MH978710	(TA)8	F:TGCACCAAAACTCAAAGCAACG	60	2	0.43	0.34	0.52	167-171
			R:TTCACCAACTGATGCCTTAGCT						
BG0445	MH978709	(GA)7	F:ACAAGATATTGACGGATGGCGA	60	2	0.33	0.23	0.45	107-109
			R:ATCAAACCCAGGAATAGGCAGG						
BG0452	MH978708	(AC)6	F:TGCTGGTTATGCATGTATGATGT	60	2	0.58	0.41	0.60	276-278
			R:GGTTTTAAAGATCCCGGCGTTT						
BG0456	MH978707	(TC)10	F:ATGGGCATTCTGTCTCACAACT	60	9	0.92	0.78	1.72	236-264
			R:GTGTTTGCAGAGTTCCATGCAT						
BG0461	MH978706	(AAAT)5	F:TGGAGGAAAAATCCAGAGAGCC	60	4	0.57	0.64	1.22	249-273
			R:AGGAGTCAGCTTTTCAGTGGAG						
BG0463	MH978705	(ATT)7	F:CCGGCTTGGAGATGTCTTTAAC	60	6	0.36	0.24	0.47	255-270

			R:ACGTTAGCAGTGTTGAGATTGT						
BG0470	MH978704	(AG)7	F:GAAACGCATCCGAGAAAGACAC	60	6	0.83	0.60	1.15	106-126
			R:GTTCCCCATCTTTGAACTTGGC						
BG0474	MH978703	(GA)6	F:GGTCATGGTTGAGGTACGAGTT	60	3	0.79	0.55	0.88	174-178
			R:GTGTCAACAGCAGAACCAACAA						
BG0476	MH978702	(AT)7	F:TGGACAGGATGAAAGCGAATCA	60	5	0.83	0.61	1.11	208-226
			R:ACGTGGATCAAACTGATGGGTA						
BG0478	MH978701	(CT)6	F:TAATTCAGCCCACCCAAAGGAA	60	4	0.71	0.68	1.23	101-113
			R:CAGGAATTTGGGTGGTTAGGGA						
BG0479	MH978700	(AG)6	F:CAGAAGGGCTAGTTTTGTTCGC	60	9	1.00	0.84	1.98	163-181
			R:GCACTACTCCTGTACACTACGG						
BG0483	MH978699	(AG)6	F:GCTGTGGGGTCTTGGTTATGTA	60	2	0.13	0.12	0.23	227-229
			R:GTCTACCACTTGAAGTTAGCTCCT						
BG0485	MH978698	(TA)6	F:TCCTAAAACGGTGTCATTTTTGAGA	60	7	0.26	0.24	0.60	269-283
			R:CTAGCCGTGCACTCCATACATA						
BG0492	MH978697	(TA)6	F:AGGATTGACTGAAGGCTACGTG	60	9	0.75	0.68	1.55	228-250
			R:GATTAGCGCTTGAAATGCCACA						
BG0494	MH978696	(AC)7	F:GGGAAAGAGAAAGGGTTGGTCT	60	5	0.67	0.54	1.04	103-113
			R:TGAAAGTTTCTACTCCCACGGG						
BG0497	MH978695	(GT)7	F:AACCGCTTGTACATCCCTAAGC	60	4	0.67	0.63	1.13	247-253
			R:TCCCTAGAGTATGAAATCTTCACAT						
BG0505	MH978694	(TTTG)5	F:CCTGTCTGCAGCATAAATACGTG	60	3	0.13	0.20	0.42	264-276
			R:GCACAAATTGGCACTCATTCCT						
BG0509	MH978693	(GA)9	F:AGACTAGCCATGATCAACGTGT	60	7	0.88	0.71	1.49	251-263
			R:CCAACTGTTGAGAACTAGCAGC						
BG0514	MH978692	(AG)6	F:GGAAGCAGCTCAAATCAAAGCA	60	3	0.71	0.52	0.90	144-148

			R:AGAAGCGTCTGTCTCAACTCAG						
BG0517	MH978691	(AT)6	F:ACTACGCCACCCTACATCAATC	60	9	0.83	0.76	1.63	112-142
			R:CTCTGCCTCGTTGTAATCCTGA						
BG0519	MH978690	(TA)6	F:AGAGTTACAAGCAGCACAAGGA	60	7	0.83	0.76	1.55	221-245
			R:TAGAAACGTTGGGAGTGGATGG						
BG0521	MH978689	(CT)7	F:TCTTGTCGGTTCAAAAACACGC	60	3	0.91	0.58	0.95	269-273
			R:TTCGTTTTCTTGGAAGTTGGGC						
BG0522	MH978688	(TC)7	F:ACGTCACTGAGTCCATTTCACA	60	4	0.83	0.58	0.99	244-252
			R:TCACACCTCGTAGGAACTTGTG						
BG0524	MH978687	(AT)13	F:ATTGCAACCACTTTAAGCGGTT	60	11	0.79	0.84	2.12	223-253
			R:GCAAGCCAATACTTTGCCTAGG						
BG0525	MH978686	(AG)9	F:ACTTCTTGGACCACAATCAGCT	60	5	1.00	0.70	1.31	257-267
			R:CAGCCTGAAAGTTGTCGAATCG						
BG0526	MH978685	(AT)11	F:ACTTAGGTCTTAACTAGGCACTTGA	60	9	0.75	0.61	1.27	202-226
			R:TCAGCACTCACTCACATGGTAC						
BG0531	MH978684	(CAA)5	F:CACTCTCGCAGATCATCTCTCC	60	2	0.50	0.48	0.68	187-190
			R:ATTGTCCCCGCTTGTTGATAGT						
BG0533	MH978683	(TCT)10	F:TCTTTCCCTTCATCTTCTGCGA	60	7	0.92	0.69	1.30	162-186
			R:CCCTTGTGAGAAGTACTGGGTC						
BG0536	MH978682	(CT)13	F:ATTGGACTTCAGGTCATCGCAT	60	10	0.96	0.83	1.96	107-131
			R:ACAGAGAGTGTGTGATGGAAATCA						
BG0540	MH978681	(ATT)6	F:TCCCCTGATTCCCTTCCACTAT	60	4	0.63	0.53	0.94	107-119
			R:CCCATTTTATGCGGTTGAATGC						
BG0542	MH978680	(TAA)5	F:AGTCGTTAAACCACATAGGCCA	60	2	0.54	0.39	0.82	209-212
			R:AGAAAGGGGCACTTCCTTATGG						
BG0544	MH978679	(AC)10	F:GAGCTGGTGTTGAGAAAGTTGC	60	3	0.33	0.33	0.63	176-180

			R:TGGAGTGGACCGATTCATCTTC						
BG0545	MH978678	(AG)6	F:CTCTGCCCCTCTCAACTGTATC	60	3	0.87	0.65	1.07	264-268
			R:TTGCTCCCACATCAGAAGTACC						
BG0546	MH978677	(ATT)7	F:TCCGAAGATGTTGGAAAACACT	60	3	0.83	0.64	1.05	170-176
			R:AGTACTCTGTTCGGCTTGGTTT						
BG0547	MH978676	(TC)9	F:GCTTCCTCCTCTCTTTCCTACG	60	5	0.67	0.66	1.23	112-120
			R:CACTCCCCCTTCATTCCATCAA						
BG0548	MH978675	(TC)6	F:TAAAGCACCCTACAGCCTCAAC	60	2	0.29	0.30	0.48	242-244
			R:GCCCAATGTTGTTAGCGAGAAG						
BG0550	MH978674	(AT)6	F:GGGGGATCCTCTCCATTTCATG	60	7	0.75	0.75	1.57	167-181
			R:TCAATCATTGGATCGGTCATTGT						
BG0551	MH978673	(GA)7	F:ATCAACAAATGCTTCCACTGCC	60	4	0.63	0.55	1.02	125-139
			R:GGTGGGTTCTCAGCACTCTATC						
BG0553	MH978672	(GA)6	F:TGTGTAGACTTTCTTTCCACCTCA	60	2	0.08	0.08	0.17	237-239
			R:AGACTGGAGGCGTATTGCTAAG						
BG0554	MH978671	(TC)6	F:TGGGAATAGCACAACAATTGACA	60	7	0.92	0.76	1.62	115-129
			R:CCGGACTGGTAGTAAAATCCCA						
BG0559	MH978670	(TA)11	F:GACGGTTGGTGACGTTGTTTTT	60	7	0.75	0.72	1.48	210-228
			R:AGCTGTCAACCACCACCATATT						
BG0562	MH978669	(AAG)5	F:TTGGTTTTCCTAGCACAAGTCT	60	2	0.75	0.47	0.66	272-275
			R:CCACCCCACATTTCAAGGCTAT						
BG0563	MH978668	(AT)8	F:AGCAGTATCCAAGACAACACCT	60	3	0.25	0.45	0.80	255-259
			R:AGGAAGCTTTACCATTCCACAA						
BG0566	MH985747	(AG)11	F:AACCACGACAGCTGAGAGTAAG	60	11	1.00	0.85	2.06	132-152
			R:TGCCTTCTTTGGAAAGGCATTG						
BG0572	MH985748	(AG)9	F:GTTTCTCTCTCTCGGTGTCGTT	60	4	0.50	0.49	0.92	253-259

			R:CTATTTTCTTCCCTCCCGCCTT						
BG0573	MH985749	(GT)7	F:CTACCCCTTACCTCAACCCCTA	60	6	0.75	0.59	1.16	220-234
			R:TGTTGTTGGGGTCAAAACTGTG						
BG0574	MH985750	(TC)7	F:CATGTCGCTAGGTCACATGAGA	60	7	0.57	0.55	1.11	199-219
			R:GGTGTGTGAGAATCGTGTAGGT						
BG0577	MH985751	(AT)7	F:TTTGCCGAATCATCAACGTGTT	60	3	0.67	0.51	0.75	133-139
			R:GACACCAAGCTAGGGAAGTTGA						
BG0583	MH985752	(AAT)8	F:AGCCAAAACACAATGGGTTTCT	60	4	0.83	0.74	1.36	101-113
			R:CGTCACACATCAATTTGGAACCA						
BG0589	MH985753	(GA)8	F:TGCTTTGGAGGCCAACATCATA	60	3	0.38	0.32	0.58	222-232
			R:TGCGAATAGAGGGCTTTCCAAT						
BG0591	MH985754	(AAT)8	F:GCATCGCCATTCTCTCTTAACT	60	3	0.46	0.52	0.89	237-243
			R:TCATGCGCCTAATTCATCCCAT						
BG0592	MH985755	(AG)8	F:ACGAGTAGACTGTTGAGAACTCT	60	4	1.00	0.72	1.33	161-173
			R:AATGGCACTAAAACCGGGTTTG						
BG0598	MH985756	(AT)7	F:ACACACACTGCCTTCTTCTTCT	60	4	0.79	0.67	1.19	169-175
			R:AGTCCAAAATTGCAACTTGCCA						
BG0599	MH985757	(TA)8	F:CACGACACAACATGCAACATCT	60	5	0.79	0.66	1.26	103-111
			R:AAATCCAGAAGGGGCTAACTAG						
BG0606	MH985758	(TC)6	F:TAATGTAAAGGTCTTCGGCCCC	60	3	0.71	0.51	0.75	117-123
			R:AGGTCCATGTCACCATGTGTAC						
BG0609	MH985759	(TG)16	F:TCGTCGCGCAGTAAAAATTTGT	60	10	0.63	0.55	1.24	189-211
			R:TAGCGGCGATTGTTCCATTTTC						
BG0611	MH985760	(TA)6	F:CGAAACGTATTTTGGAGCATATGC	60	3	0.33	0.30	0.55	196-200
			R:CCCCCTGAAGATTTTCGCATTG						
BG0615	MH985761	(CT)7	F:AACCCTATAAAACCCCGACGAC	60	2	0.17	0.15	0.29	120-122

			R:GGAGGGAGGAGGAGAAGTTAGT						
BG0616	MH985762	(AT)6	F:AATTGGTCAGTCTTCAGGAGCC	60	4	0.96	0.64	1.15	197-205
			R:AGCATGTCCCTCACTTATGTGG						
BG0618	MH985764	(TA)9	F:GCTGGATTGAGAGTCAGGATCA	60	9	0.67	0.82	1.90	248-264
			R:ACGAAGACGAGAGAGAGAGTGA						
BG0620	MH985763	(AG)7	F:TCCCTTGGAAAAAGCTTTGGGA	60	2	0.08	0.08	0.18	283-285
			R:GATGATATGGCCCACCCTCAAA						
BG0625	MH985765	(TC)9	F:GTCTCCAAAACCAAGCAAAGCT	60	6	0.88	0.66	1.30	213-225
			R:TAAAACAGCTGAGTTCCCGAGT						
BG0631	MH985766	(AG)8	F:TCTCCACAACGACCCTTTACTG	60	3	0.04	0.41	0.70	218-224
			R:GCTTGTTGAGAAGGCATTTCGA						
BG0637	MH985767	(TA)7	F:ACCAGACGGTCTGCATTAACTT	60	5	0.79	0.57	1.00	125-137
			R:TGTCGATACCTTTTCACTCATCCA						
BG0640	MH985768	(GA)7	F:CAATGTGGGCATGGCATAGAAG	60	2	0.67	0.44	0.63	155-157
			R:CTTCTGCCTCTCTCCGTTTCTT						
BG0648	MH985769	(AAT)5	F:TGAAACAAATGGAGCTTTAGGGT	60	2	0.54	0.50	0.69	131-140
			R:GAGCTATGCCATTTGGATTGGG						
BG0655	MH985770	(TA)6	F:ACTTGGGTGTTTATGCTTTTTCTGT	60	2	0.67	0.44	0.63	184-188
			R:GTTGAGGAACCTAACAATCTATCCT						
BG0657	MH985771	(GA)6	F:TCAACAAACGCGGCATAGATTC	60	2	0.58	0.41	0.60	175-181
			R:ACTCCACCGATTCCCTGAATTC						
BG0660	MH985772	(AT)10	F:CCCCTTTATCCTGAGTCCAAGG	60	8	0.92	0.79	1.75	233-247
			R:GCCCCCAAATTAGTTGTTGCAT						
BG0661	MH985773	(AG)7	F:GGCCTATAGCAATGTGGGTGTA	60	2	0.29	0.25	0.41	196-198
			R:ACATTTTCACACCTCTCCTCCC						
BG0667	MH985774	(AT)7	F:GCATTGCGTGGAAATGTTGGTA	60	2	0.33	0.39	0.77	174-186

			R:GCCAAATTGTTAGCCCCAAAGT						
BG0683	MH985775	(TTA)6	F:AGCCAAGTGTTATAGCAGACTT	60	9	0.92	0.84	1.96	226-250
			R:AGAAGATGTCCAAGAACGTGCT						
BG0684	MH985776	(CT)11	F:TCCTCTTCTCCACAAGCAAGAC	60	17	1.00	0.88	2.39	226-262
			R:TCTTGCCTCCCCATTTCAAGAA						
BG0685	MH985777	(GA)7	F:TTTCTGCTTCTTTGCCGTTCAC	60	6	0.92	0.62	1.14	101-117
			R:CACGAGCTGCATCTCTCTCTAG						
BG0687	MH985778	(TA)6	F:TCTATTATAGGGGGCGGGACAT	60	7	0.79	0.64	1.35	173-191
			R:GGAGGTAGAAGCTCAAACCCTC						
BG0693	MH985779	(AG)13	F:ACCCTCCTAAGTTACACCAGAC	60	8	0.92	0.85	2.01	214-230
			R:GGCACATGTCGAAGAAGCTACT						
BG0694	MH985780	(TC)8	F:GTCCCACTAACGTGATACTCCC	60	2	0.21	0.19	0.33	101-103
			R:ACGGAGGAAATGGAGAGAGAGA						
BG0695	MH985781	(AGA)5	F:AGTCAACTACGTTGTCCACTCC	60	2	0.50	0.37	0.56	234-237
			R:CTGCTTTTGGCTGACTTCCATC						
BG0696	MH985782	(TA)8	F:TACATGGGTGTGCGAAAGATGA	60	5	0.96	0.64	1.17	158-166
			R:ACTGTACGCAAATGTAAATTGTTCA						
BG0700	MH985783	(AT)7	F:AAGTTTGTCTATCTCTCTACACACA	60	4	0.92	0.67	1.22	260-270
			R:GCACACTTCCACGCTTTTCTAC						
BG0713	MH985784	(AT)8	F:CAACAAATTGGACCATGAGGGC	60	6	0.88	0.78	1.67	143-153
			R:TCTGTTCTCCATCAGTATGTTTGT						
BG0714	MH985785	(GA)7	F:AAAGCGATACCGAAATTCGCAG	60	10	0.83	0.77	1.79	101-123
			R:ATTATTCCCACCGTCACAACCA						
BG0719	MH985786	(GA)7	F:AGACACGTGAAGTCAACAGTTT	60	4	0.42	0.43	0.79	168-174
			R:ACTCTGGTTTGGCCACTCTAAG						
BG0723	MH985787	(TATTTT)5	F:GTAGGTAGGCCCATGTCTCATG	60	3	0.50	0.62	1.02	215-227

			R:ACAATTGTTGCACCTTCCACTC						
BG0732	MH985788	(CA)8	F:AATTGGCTCCTGGGAACTTCTT	60	5	1.00	0.72	1.36	154-162
			R:TCCCAGAGTTTTGATGGTGATGA						
BG0736	MH985789	(AG)6	F:TCAAAAACTCCAACCCAAGATTG	60	2	0.33	0.48	0.68	104-106
			R:TCCCCTTTGCATGACACTGTTA						
BG0738	MH985790	(AAT)5	F:TCTAACAAAACCATGTTCCCACT	60	3	1.00	0.56	0.89	202-223
			R:GTTGACTACTTCGCTGATATGCA						
BG0742	MH985791	(TA)9	F:TCGAGACACACTTAACAAATGGT	60	10	0.92	0.77	1.71	240-266
			R:AGAGAGAGAGAGAGGCGAAACT						
BG0743	MH985792	(TC)7	F:CTGATTGTGACTACGCCAACAC	60	5	0.75	0.78	1.56	262-270
			R:CAAGTCTGATGCATGCTAAGCC						
BG0745	MH985793	(AG)8	F:TGTGTTTGATTTTAGTGCCTGCA	60	3	0.54	0.53	0.90	203-207
			R:AAAGACAATCATCACCACCGCT						
BG0748	MH985794	(AT)6	F:GGTGGAAAGGTAGGCACATACA	60	3	0.79	0.19	0.40	231-235
			R:GACAGCCTAGTCCTAGTGATGC						
BG0749	MH985795	(TCT)6	F:TGAAAAGGAGTCCAACAACAGAGA	60	5	0.92	0.70	1.36	135-153
			R:GAGGAGAAGGCTCATGGGTATG						
BG0750	MH985796	(TA)10	F:TTTCTGCCATATTCTGACGTGT	60	3	0.42	0.34	0.57	151-157
			R:TTGTCCACTCCCCCATACATTG						
BG0751	MH985797	(TA)11	F:ACCCATGGTTATGAGCGAAGAG	60	11	0.63	0.57	1.39	224-248
			R:CATATGCACAGCACAGTACTGC						
BG0756	MH985798	(CCA)5	F:CAACCCACCTAGAAATCCACCA	60	3	0.83	0.56	0.90	188-194
			R:TGCCAAGAAAGGTGGTGTTATTG						
BG0758	MH985799	(TC)13	F:GTTGTTGAAGACAGATGGTGGC	60	10	0.79	0.83	2.02	192-230
			R:GAACTCAAAACCCGTTCTCTGC						
BG0759	MH985800	(TG)15	F:TGCTCTATGAGGTGGTTAGCAT	60	3	0.13	0.14	0.32	238-244

			R:GATCAAAAACCCAGCATTCCCC						
BG0761	MH985801	(AT)15	F:ATCTCACCTAACATCCGCTTGC	60	12	0.92	0.81	1.88	126-154
			R:GGCCTCATCTTGACCCTCATAG						
BG0763	MH985802	(TAT)16	F:AGTGGGGCTTCGTTCTTTCTAG	60	7	0.92	0.73	1.54	177-195
			R:GCTGTATGTTTGTCGGTTTGCT						
BG0765	MH985803	(GA)6	F:GACTAAATTTGCTGGCCGTACC	60	3	0.79	0.49	0.73	236-240
			R:CAATAGCTCTGACCGATCGGAT						
BG0767	MH985804	(CT)8	F:AGCCTTGAAACTGGAAATTTTGTCT	60	5	0.50	0.66	1.27	181-189
			R:CCCCATCCTCCCAACCATTAAA						
BG0768	MH985805	(AG)9	F:ATTGACTTAAAGCTGACCGGGT	60	8	0.92	0.79	1.80	209-225
			R:CTAGCTGTATTTCTCCGACCCC						
BG0771	MH985806	(AG)10	F:ACGGCTCTCATTTGACACAATA	60	6	0.88	0.60	1.10	172-184
			R:GTTCTTGGCTCACAGAGGAGAG						
BG0773	MH985807	(AT)6	F:ACTCTGACACCCACTTCTAGGT	60	4	0.96	0.61	1.08	124-134
			R:AGCCTTCATGCTGTACTCACAA						
BG0779	MH985808	(CT)9	F:ATCATGTTAGAGCTGGTCGGTG	60	3	0.63	0.54	0.87	256-262
			R:CACCCTGAGCAACACAATTTGT						
BG0780	MH985810	(CT)6	F:GTCCTACATCAAGGGCCAGAAT	60	4	0.88	0.57	0.98	270-282
			R:GCATTGCTCTCAAGTCTCCACT						
BG0783	MH985809	(AAT)6	F:CGAATCTTGTGTTCGGATGCAT	60	4	1.00	0.65	1.12	269-284
			R:ATGACCTCTTTCTTCAGCAGGG						
BG0786	MH985811	(AAG)7	F:TGACATTACCCGTGTTTTTGCC	60	5	0.71	0.54	1.02	215-227
			R:AAACCTTTCAGCTTCATGTGCC						
BG0790	MH985812	(TC)6	F:GTCAGGGAAAAACACATTCCGG	60	4	0.71	0.66	1.18	100-116
			R:TATCACCCCTAGGCAATCCTCA						
BG0793	MH985813	(AG)14	F:ACCAAGAGAGAAATCTTCTAGAACA	60	11	0.75	0.88	2.25	163-187

			R:CCGCCATTGTTGTTTCGTTACT						
BG0797	MH985814	(TC)6	F:AAAATGGTCTCCCCTGACTTCC	60	6	0.96	0.70	1.44	166-182
			R:TGGGTCTGGTTTCATGCCAATA						
BG0798	MH985815	(TC)6	F:GCCGTATTGCTGTCTTCTTCAC	60	3	0.50	0.48	0.72	181-187
			R:AAGTTAAGAAGCCCCTCACGAG						
BG0799	MH985816	(AT)12	F:AGCCTGATTAGCCCCCATAAAA	60	6	0.63	0.74	1.56	249-261
			R:TCCCCAATGGTCATGGCAATAA						
BG0802	MH992132	(AAG)5	F:CCTGAACACACAACAATGCGAT	60	5	0.58	0.66	1.27	251-263
			R:CTATCACCCAGATCGTGCTGAA						
BG0803	MH985818	(AT)7	F:GCGCTAAGAGTGAGTGAGAGAA	60	5	1.00	0.65	1.24	242-258
			R:CGCTAATCGTAAAACCGCCTTT						
BG0806	MH985819	(CT)10	F:TCAGCTTATGCAGGCCTAAACA	60	8	0.88	0.74	1.67	253-269
			R:TATGACAAATGGAAGGCGGACA						
BG0810	MH985820	(TC)9	F:AAATCTGTGAACAAGTGCTGCC	60	4	0.50	0.44	0.85	140-146
			R:AGGCTTGGCTTTGATACACCAT						
BG0813	MH985821	(TTC)5	F:CGAGTAGACTAAAATCGCGGGT	60	3	0.42	0.35	0.65	224-236
			R:CCGTATGCTGATGTGTTGAGAC						
BG0816	MH985822	(AT)6	F:ATCAGGTTGACAGTGCTGACAT	60	6	0.92	0.79	1.61	159-173
			R:AAATGTGACATGTTTCCGAGGC						
BG0818	MH985823	(TC)11	F:GGAGATGCAGAGGGATCGATTT	60	7	0.92	0.76	1.57	210-232
			R:CAGCCAAACAGTCGAAACGAAA						
BG0824	MH985824	(AT)6	F:TCACCACTCCCATATGGCAAAA	60	5	0.29	0.51	0.92	162-188
			R:TTGTCAAATGTCACAGCAGCAC						
BG0826	MH985825	(AG)7	F:CGAGTAGACTATTTTGATTTGTGCC	60	4	0.54	0.49	0.91	136-146
			R:TATCAAGGACTCTGCCCAAACC						
BG0828	MH985826	(TAA)12	F:GCCCGCTTGATAAAATGTCACA	60	8	0.46	0.81	1.83	250-274

			R:TTTGATTCCAGGCGTTCTTTGC						
BG0831	MH985827	(TC)6	F:AGGCAAGGGACGTTAAGCTTAG	60	7	0.75	0.66	1.32	204-218
			R:GGTAACCTGCTAAGAGGTGAGG						
BG0833	MH985828	(ATT)6	F:AGTTACCGCCGCCTAAAATATT	60	3	0.75	0.50	0.84	261-267
			R:AGAGCCAATAATAACCATTCTTGCA						
BG0842	MH985829	(AG)6	F:AATAGACCCTCCTACGTCCCTT	60	2	0.21	0.25	0.41	247-255
			R:CGTTCGATGACTGCCCAAATAC						
BG0847	MH985830	(TC)6	F:ACTTTAAATTGTGGATGGGTCTCA	60	4	0.21	0.20	0.46	208-218
			R:GCTGATCACTGTTTATCATCGCA						
BG0848	MH985831	(CT)6	F:AGTCAATTGTAGGGGCCGAATT	60	5	0.75	0.65	1.19	138-152
			R:AACACCCTCTTACAAGCTCTCG						
BG0849	MH985832	(GA)6	F:TGCCTACTTCCAAATGACCGAA	60	6	0.96	0.74	1.55	179-195
			R:GACGCGACTCGATGTTAATACG						
BG0852	MH985833	(AT)9	F:CCACCAGGGTAGAAAGCTATGG	60	7	0.92	0.80	1.69	129-143
			R:GCTTCTTGAGGTCATAGTTCAACG						
BG0854	MH985834	(AT)8	F:TGTCATTCAGCCCAAGCCTAAT	60	10	0.21	0.78	1.74	145-175
			R:CCAAAATCTTGGCCCAAACTCA						
BG0856	MH985835	(AT)7	F:GAAACTTGGGTGAATCCACGTG	60	3	0.63	0.59	1.19	236-246
			R:TCAGGTGCATAGCATGTGACTT						
BG0864	MH985836	(GA)8	F:ATTGGTAGTTGAAGTGTTGCGC	60	6	1.00	0.76	1.52	204-214
			R:CCTCCAGTGCCTTATCTGAGTC						
BG0866	MH985837	(CTGCCC)5	F:CTGGTGCGCTACATTGTTCTTC	60	3	1.00	0.65	1.07	165-177
			R:TTCCCTAGGTTCCCGAAAACTC						
BG0872	MH985838	(TTA)8	F:TGATGGAGTTACGAGCTGCTTT	60	5	0.46	0.74	1.46	220-235
			R:TACCCTAGCCCCCAATATGACT						
BG0876	MH985839	(AAT)10	F:TGGGCTGATATGGTAGTGTTCA	60	6	0.54	0.61	1.16	115-130

			R:ATATTGCATTGGGCTGATGTGC						
BG0878	MH985840	(TA)8	F:CCTGTGACGATCGGTTCAATTC	60	5	0.38	0.37	0.77	127-137
			R:CCCCATTTGGTAGAGAAGACCC						
BG0879	MH985841	(AG)13	F:GGAGTTAGAATGGTGCATTTGACA	60	13	1.00	0.89	2.34	215-239
			R:ACCCTAACAATAGCGCACATGA						
BG0880	MH985842	(CT)8	F:TCAAAGACCCAGCTGTGTCTTT	60	3	0.75	0.60	0.99	221-227
			R:CCTCTCACAAAACTCATGCTCC						
BG0881	MH985843	(AGG)6	F:TGGACGACAAGACTGCTGTTTA	60	8	0.50	0.66	1.48	169-196
			R:CAGGAATCCCAACTTCCCCTTT						
BG0883	MH985844	(AAT)9	F:CCCATGCATGCTCAATGTAGTG	60	4	0.13	0.68	1.26	251-260
			R:CACATGCCATCAAACGTGATCA						
BG0884	MH985845	(AT)8	F:AGTGATGGAAGTTCAAGGAGGT	60	5	0.67	0.50	0.93	230-238
			R:GGCATGACCCACTACTGTGAAT						
BG0885	MH985846	(GAT)6	F:TGGTCTGATGCATGAGTACCAA	60	3	1.00	0.66	1.09	262-268
			R:GGCAAGAGAACAGGCAGATTTG						
BG0887	MH985847	(GA)6	F:CCAACTGAACATGCTTTTATTTGCA	60	2	0.17	0.15	0.29	204-210
			R:CCATAAGCGACACTGAGCCTAT						
BG0888	MH985848	(ATTT)5	F:ACGTCACAGGTAAATACTGCAA	60	4	0.88	0.74	1.37	263-279
			R:GGGTGCCAGTGTGTGTGTAATA						
BG0889	MH985849	(TTG)5	F:GGGAGGGAGAGAGACTGTCATA	60	4	0.92	0.60	1.02	275-287
			R:CCAGAGCCAGAATCCAGAATCA						
BG0891	MH985850	(TAT)8	F:TGATACACACCAGCATGTTGGA	60	9	0.67	0.82	1.88	139-166
			R:CGTACTGGCCATTTGCTTCTTT						
BG0893	MH985851	(ATT)9	F:CTCGAGTCAGCATGAAGGTCAT	60	6	0.96	0.82	1.75	247-265
			R:TTTGGAACCAGATCCTTCGGTT						
BG0895	MH985852	(AT)6	F:AAGCGTGCAACCCACTAATTTC	60	3	0.88	0.52	0.78	188-192

		R:TATAAGCTGCTGTTCGGATGCA			

Note: Ta, annealing temperature; N_A, number of alleles; H_O, observed heterozygosity; H_E, expected heterozygosity; *I*, Shannon-Wiener diversity index.

Table S2. The PCR results for 270 SSR primer pairs in 24 samples of *Betula alnoides*.

Primer Name	Motif	Primer Sequence	Ta (℃)	N_A	Size of allels (bp)
*BG0001	(ATC)5	F:GAACAGGTGTTGAGCATGCAAT	60	1	245
		R:TTTCGCCTTCTAAAACACCCCT			
○BG0003	(TC)8	F:CTTCTGATGGCGTGGTATCTGA	60	/	/
		R:CGAGATCGTTAGAAGCTCTCCC			
*BG0012	(ATT)5	F:GCACTTAAGAGGATCCGAACCT	60	1	161
		R:ATAATTTGGAGCGTTGCACGTC			
*BG0017	(AGA)6	F:CTGCAGGTGAAAGCATTTTCGA	60	1	244
		R:TTACGGTTGAGTTGCACGAGTA			
○BG0022	(TA)13	F:GACTACGAGTAGACTAGCACTTTGA	60	/	/
		R:ACATACCTAACCAATGTAGGACAT			
○BG0031	(TA)7	F:ACGAGTAGACTATCCTTCGCTT	60	/	/
		R:TCAACTACAGCTCAGCATGGAG			
*BG0038	(GA)7	F:TTTGTTTCAACCATCCTTTGCA	60	1	271
		R:ACGTCTCTCTCTCTTCCTCGTT			
*BG0042	(ATT)5	F:GGGCAATGTAGGAGTTCCAAGA	60	1	138
		R:GAAGCTTGGGTTTGATGGTCAC			
○BG0045	(AT)10	F:CAAGTGGAAAGCTAGGGTCTCA	60	/	/
		R:TCCGATATAGAACTCAATGCACACT			
○BG0052	(AG)9	F:AAGCCAAGCTAAGTTCCAATGC	60	/	/

		R:TCTACTAACGTGGACCTCTGGT			
○BG0056	(AT)6	F:ACCCCCTTTATTTGGTCTCACC	60	/	/
		R:GACATGCAGTCCTGTCGTTATA			
*BG0057	(TCT)6	F:CAGCTTTTTATGCTTGGCTGGT	60	1	168
		R:GTAAATGGCTGCCAGGATTGAC			
*BG0059	(CA)6	F:TGGGTAGCTAGAAACAACTTAACCA	60	1	238
		R:TCAAAGCTTGAGCAAAGATGGC			
○BG0060	(TA)9	F:GCTAAGCCAATCTTGTCTATGCC	60	/	/
		R:ATAGGACTCCTCCACACATGCA			
○BG0063	(ATT)5	F:AACAACAGCCGTCACTTCTTTG	60	/	/
		R:GCATTGCTTTGGGTTGGTGATT			
○BG0067	(TA)10	F:ATCAGAGATGAGGGAAGTGGGA	60	/	/
		R:TATCTAGGGCTGAAAATGCGGG			
*BG0068	(TTA)5	F:TGGGCTAGGATCTTCTCATGGA	60	1	280
		R:TCAAACACCTGCAGAAAGACAA			
○BG0072	(AG)10	F:ACGCTCTGGAGAAAACTACTGG	60	/	/
		R:CGAGAAACGAGCTAGGAAAAGG			
○BG0081	(AT)6	F:CCCACCAGGTGTGTGTTTAAAC	60	/	/
		R:GGCAATGGCGAATTGTGTATGA			
*BG0084	(ATT)7	F:GAGAAGTTGCTTGTGCGGAAAT	60	1	124
		R:CAGAGGTACATGATGCCTGAGG			
*BG0092	(AC)8	F:TCAAAGTTTGGGGGTATGGGAA	60	1	240
		R:GCTCGTGGCAATTCGAATTGAA			
*BG0093	(AG)6	F:TCGGTTAATTCGGTGTCGGTAA	60	1	273
		R:CTCTCAGTCTCTCACAGTCACG			

○BG0094	(CTT)6	F:GCAAAAAGGCCTCATCAGCTTT<br>R:TCTGAGATTCCGGCGTTATCTG	60	/	/
○BG0103	(TA)12	F:GCTGCCGCATCTACCAAATTAG<br>R:TGCAGACATTGTTCGATTGTTGT	60	/	/
◇BG0104	(AAT)5	F:GACCAAGCCACCTCATGACTAT<br>R:TAAAGGTGGCCGAACAATCTCA	60	2	127-133
○BG0105	(TCT)8	F:CTTGTAAGGACTGTTGCAACCC<br>R:AAGGCAAGCTGAAGATTTGCAG	60	/	/
◇BG0109	(ATG)7	F:AGTAGACTGACTCCTGCCATCT<br>R:ACTCTATTGCTTCTTGGGCGAA	60	4	129-141
◇BG0110	(TA)6	F:TGATCTCAGTGCTCAAACACAGA<br>R:ACGTGGCAGAGACTTTATGGTT	60	3	241-247
*BG0130	(TC)8	F:GAGTAGACTGTCTCGCTGAAGG<br>R:ACGAAGACCACGCTCTTTGTAT	60	1	145
*BG0135	(CT)8	F:ACTCCTTTGGCTCCTGTCATTT<br>R:TGGTGTAAGAATGGTTGTTGCA	60	1	236
○BG0137	(TA)7	F:GCAAATTAACCGTTAACCGCCT<br>R:CGGTTAGGGTTTAAAACGGTGG	60	/	/
*BG0138	(AAGA)5	F:AACTCACCATTTGACTGCAAGC<br>R:AGGTAACGGTTGTTTGAATTCCA	60	1	267
○BG0140	(TA)8	F:GCGTGGTAAAAAGTCTGCTGTT<br>R:TCATGCATGAAATTAAGCTGACCA	60	/	/
○BG0152	(TA)12	F:AGCCCCTCTACATGTGAAATCA<br>R:TCTCATGTAGAGGGGTCAAAGT	60	/	/
○BG0160	(TA)6	F:TACGAGTAGACTGGCTTAGGGT	60	/	/

		R:CGATGGTGAAGTCAGTTGGAGA			
*BG0168	(ATA)6	F:ATTTGCTCCCCCATAAGAACGT	60	1	265
		R:AGTCACCCGTTTATGGCTCATT			
*BG0170	(TA)6	F:GTTACGCCGCTGTCCTTAAAAA	60	1	105
		R:AGGAAAGCAAGTGGTGGATGTA			
○BG0178	(AT)6	F:GAGTAGACTGTAGCACCCGATG	60	/	/
		R:CGATCCACTGCATCAACACTTT			
○BG0185	(AAAC)5	F:GACCTCTCCAGCTTGCTTCTAA	60	/	/
		R:AGCCAAAGATGGATCAAGATGGA			
*BG0191	(AT)6	F:ACGCTCAATTTATCAAGCTGGC	60	1	254
		R:CCCTCTCGTCTCCATCAAAACA			
*BG0192	(TC)6	F:TTCCTCCCTCACGACATCTTTG	60	1	122
		R:GGAATGAGAGATGCCTGAGAGG			
*BG0194	(TG)6	F:GCCATTGAGGTAAGATTTGCAGT	60	1	280
		R:GTGCAAAAGTACAACCCTGGG			
*BG0195	(ATG)5	F:TGGGCTATGATGTGAGAATGGA	60	1	275
		R:ATTAAGACAAGCACATGCCTGC			
*BG0196	(ATA)5	F:AGCTGCTTCCTAACTAGTTTCCT	60	1	274
		R:ACCTTTTTGCTTCCCCTAGACA			
○BG0197	(CT)8	F:GTAGACTCCGTGTTTGGTAGCT	60	/	/
		R:CACGATTTTGGCCCTGGAAAAT			
○BG0199	(TA)6	F:AAGATTATTGCGCGCGACATAC	60	/	/
		R:AAAAGGCGTGCCATGTGTTATT			
*BG0200	(GA)6	F:ACAGATGCTTCATCATCGTCGT	60	1	210
		R:GATTCGGACTCGGGAACTCTAC			

○BG0204	(TA)16	F:GTCCCACGTAGCCTAGTTTGAA<br>R:CCTTGCCTTATGATGCTGTTGG	60	/	/
*BG0209	(AAAT)5	F:ATGAATATAGGGAGGCCAAGGG<br>R:TCAACAAGAAGCAGGTCAAGGA	60	1	215
*BG0210	(ATT)6	F:TGGGTTACCTGAAGAACATGCA<br>R:TCCTCCTTTTTGGTGTATCATCT	60	1	129
○BG0213	(AG)7	F:AAAGTCAAAAACTCAGGTGGCG<br>R:ACAGCACCTGGGTTTGTCATAA	60	/	/
○BG0214	(AT)12	F:CCCTGGACCTTTTGTATGGAGA<br>R:CGATTTTGCCCCACTTTGTGTT	60	/	/
○BG0215	(TA)10	F:TATTGCCACATTCTCCGCAAAC<br>R:CCATCACTTACTTTGAAGCACACT	60	/	/
*BG0223	(CT)6	F:CCCATGAACCCAAAGACCCATA<br>R:CAGAGAGAGACAGAGCCCTTCT	60	1	237
○BG0224	(TTTA)5	F:TTTTGACACGGAACAACCACTG<br>R:TCACGAGGAAAATAGTATCGCA	60	/	/
◇BG0227	(GA)6	F:TGTGCCAAGAATTGAAGGGAGA<br>R:GGCCCGATAAAGTCACCATTTT	60	2	270-272
○BG0228	(TC)8	F:TTTACGCTTGCAACACAGTCTG<br>R:GAATTTGCGGAACACATTTGCA	60	/	/
*BG0229	(GTG)5	F:TCGATATCGAGCATTGCCTCAA<br>R:CCCTCTTTATCAGCCCCCAATT	60	1	242
*BG0230	(GGCAA)5	F:ACGCTTAGAACAAGAGTGACGT<br>R:CGACCAAGATTGCCACACTTTT	60	1	157
○BG0231	(AT)8	F:TAACCTAGACTCAGCCACTTGC	60	/	/

		R:TTTGACGGATTGCTCCATACCA			
○BG0253	(AT)6	F:CGAGTAGACTTCGAAGACCCAA	60	/	/
		R:GGCTCACTAGTCAAGCGAAGTA			
○BG0255	(TA)6	F:AGAAGAGAAAAGAAGCCTGCAGA	60	/	/
		R:CGAATCGGCAAGTTACGTTCTC			
○BG0257	(TC)7	F:TTCCCAAGTTGAAGTCGATGGT	60	/	/
		R:GAGAGCATCACAAGACAGGGAA			
◇BG0268	(CCA)7	F:AAATCAAAAGCTAAGCCCGCTG	60	2	252-255
		R:TTTGGTTTCTGGTGGCTTAGGA			
◇BG0271	(TGA)5	F:TGATGAAGTTGATTGCTCTCAAG	60	3	248-254
		R:GACCTTGTGTTTACACCTTGTGA			
○BG0277	(AC)17	F:AATAGCCACCCCCGAAATCTAC	60	/	/
		R:TGGTGTGGATTAGTCAACCCAT			
*BG0281	(TC)6	F:TCCTTACACACTTCCACCATGC	60	1	116
		R:ATCCATCAGCCCTTTATGTGCA			
◇BG0298	(TA)8	F:AGACTTGAGTATTTTCCATAGGCGA	60	2	266-276
		R:GGCGCGGCTCATTTACATTTAA			
○BG0300	(GA)9	F:GAGGGGCTTGGGTTTTTCATTT	60	/	/
		R:GGCCTTTAGAGCATTTTCAGCA			
*BG0308	(AT)6	F:AATGGATGTAGGCTTACCACAT	60	1	129
		R:AAGCAACACAACGCTTCAGATT			
○BG0309	(TA)6	F:TAAGCACATGAATTCACGCCAC	60	/	/
		R:GCATAGCGCGGGTTATATGCTA			
*BG0313	(CT)7	F:TCTTGGATAATCCTGCAACGCT	60	1	240
		R:TTGCTCTGTTTCAGACCTGTGT			

*BG0319	(TTA)5	F:CCTGAGAGTCTGAAACTTTATTTGT	60	1	151
		R:TGCTAACAAATCCTCCTTCTCCA			
*BG0321	(AT)10	F:ATCACTTGTCTGCATGCGATTG	60	1	269
		R:AGGTTCCTTACACTTCTGGCAA			
○BG0322	(TAA)7	F:ACAATGCTTAACAATGCATGCA	60	/	/
		R:TCGACATCCGACTTTGAGCATT			
○BG0323	(ATT)8	F:CCTGGCTCTAGCTCGGATAAAG	60	/	/
		R:CCCTGAGGAAAGATTTCGGGAA			
◇BG0326	(TTC)5	F:ATAAAAAGACCCATGGCCGGAA	60	2	143-146
		R:TCTCCGTCTTTTCTCCTTGCTC			
*BG0332	(AT)7	F:AGTGACTAAAAACTCCTTGACACA	60	1	270
		R:AGTACCCATTGCCTTAAGGGTG			
*BG0336	(TA)12	F:TATGGTTGGGGCAATTGCTAGT	60	1	222
		R:AGGAATTCCAGCGATCACAAGT			
*BG0341	(AT)6	F:TTCTGGAGGGGAGTCTCGTTAT	60	1	252
		R:ACTCTCTAATGCAAAACTCGGC			
*BG0343	(TC)7	F:TTCCGACACCGATTTAACCGAT	60	1	168
		R:GTAGGCAAGAAGAAACGTCGTT			
○BG0348	(GA)10	F:TGGCTCATAAACCACCAACCAA	60	/	/
		R:AGAGAATCTGGAGCCCCAATTG			
*BG0351	(CT)6	F:TACGAGTAGACTCACCTCCTGG	60	1	145
		R:AAAGAGCCATCAGAATGTCCCC			
○BG0352	(TTA)6	F:TGCAGTTTGTTAGAACATTGTTACG	60	/	/
		R:TCCCATAACACTGATACGACAATC			
*BG0353	(CT)6	F:TTATTCCTCCTATGGTCCGTCC	60	1	177

		R:CCTTCTGGCCACTATGCAATTG			
*BG0359	(TA)7	F:GCATCAGCTCAAAAGACATGCT	60	1	230
		R:GGTTGTTGCCCAAACCTTAAAGA			
*BG0360	(CCT)5	F:CACGTTATTTGCACACTCAGCA	60	1	249
		R:TATTCTTGGCGTTGCTTTGTGG			
○BG0361	(CT)8	F:CTACGAGTAGACTACCGCGAAG	60	/	/
		R:GTGCCAGAGAGAAAATGGGGAG			
◇BG0365	(TTAT)5	F:TTTCCTTCCGAGTCCCTTCTTG	60	2	276-280
		R:CATTGGTTTTATCCTGCAGGCC			
*BG0371	(AAT)8	F:CAGGGTGATTGATGTTGGAGGA	60	1	243
		R:TTCAAGTTTGGAGCTGGAGGAG			
*BG0372	(AG)6	F:TGGAGGAGGATGTTGGTTCAAG	60	1	102
		R:GAATCGGGTCATCACACAAACC			
*BG0378	(AAT)5	F:GGTTGGCCTGGACTTTAGATCA	60	1	233
		R:GCATCGAATGTGGTTGATGGTA			
*BG0382	(AT)14	F:AATTAGGCCTGGGCTGAATGAA	60	1	235
		R:TGAGGAATTTTGGGTTTAGCTAGGA			
*BG0388	(ATT)5	F:CCATCCCTTGGTTTCTCTTTCTC	60	1	256
		R:CCGGTTGAATGCGTAAGGTTTT			
*BG0389	(TA)6	F:AGGGGTCTGAGACTTTTGCATT	60	1	106
		R:AGATGGGGAGGGATTAGTCACA			
○BG0390	(AAT)5	F:TCTAGCTAGGGGTGCATTCTCT	60	/	/
		R:GCATCAGAAACACAGCAGAGAC			
*BG0401	(AT)6	F:TGACCTTGGCTTGTTTGGGTAT	60	1	212
		R:GCTAGCAGCTATGATTGCCAAC			

○BG0404	(AC)9	F:TGTGTAACTCAAGTTTGGGGGT	60	/	/
		R:CAACATTCTAAGCTCGCCCATG			
○BG0405	(ATT)6	F:GCCCAACGTACGTCACTAGTAA	60	/	/
		R:TTGGGGGTTCAGGCTTATTTCA			
○BG0408	(AT)11	F:AGGCATCTTGAAAAGCTATGCT	60	/	/
		R:GGAACCTCCATCTTGAGAGAGA			
*BG0409	(AT)9	F:GCACAGTTGGAAGGGATTATGC	60	1	233
		R:ATGTGCACATGACAGCTTGAAC			
○BG0410	(TC)12	F:TCCTCTCCTTCTCTCACAGTCC	60	/	/
		R:GAAAGATCGACGGAGACAGTGA			
○BG0417	(AT)12	F:ATACAGAACCGTCAGATGGCTC	60	/	/
		R:ACTTCGTTAAGGCCCGTTCATA			
*BG0418	(AT)12	F:TGGGCTGATGTGAATCATGACT	60	1	140
		R:ACCCCCAAAATAGAGCACTGAT			
○BG0420	(TG)6	F:ACGAGTAGACTGAATAGAATGCA	60	/	/
		R:AGACCTGGAAGCAATAATGGCA			
*BG0421	(TAA)5	F:ACCTAGTCCTAATTTGCGAGCA	60	1	247
		R:CCTCCCCGGTCTCAATATTCAT			
*BG0423	(AT)6	F:CGAGTAGACTAGAGGGAGGGAG	60	1	195
		R:AGTGTGAAAGCAGTGGAGACAA			
*BG0424	(CT)7	F:TCGACTACTCCTCTTCCCCATT	60	1	118
		R:CTCTTGCACGAGAGGGAAGC			
○BG0425	(AT)6	F:AATTCGGTTGCCAAATAAGCCC	60	/	/
		R:TTAGGCTTAGGAGTTGTGCACC			
*BG0428	(GT)7	F:TGTTCAACATTTTAAGCTCGCCA	60	1	250

		R:GAGGGTATGCTACGAGCCATAC			
*BG0431	(GA)6	F:CAGAACTGAAGAGGATAGCCGG	60	1	128
		R:CGAAGCAGTTAAGGGCAGTAGA			
*BG0435	(TA)11	F:TCTGCCACCATCCATTCACTAC	60	1	200
		R:TGAAAATTAACCACCCGTGTGC			
*BG0439	(TC)7	F:TTGGGGCAAAGAACCAAAAAGG	60	1	267
		R:AGTTTCATTCATCACGCACAGA			
*BG0440	(TGT)6	F:TTGCATGCCATTGAGAAGCTTC	60	1	226
		R:CTGCACTTACAGTTGCTACAGA			
*BG0442	(TC)6	F:TGGCTAATCCATTCACTGTCATCT	60	1	242
		R:TGCATCTAGCCATGGATATGAA			
*BG0446	(AT)6	F:ACTACGAGTAGACTAAGCAAAATCA	60	1	188
		R:TTTTGAGCTTGGCTGCATTTGA			
○BG0451	(CT)7	F:AAGCATCTTCAACAAACCTTTCA	60	/	/
		R:CTTGGTGGGTTTGGTGAAGGG			
*BG0455	(TA)6	F:CTTCAGAAGCGCACTTTCTTGT	60	1	169
		R:GAACTGAAGCTGCAATGAAGCA			
○BG0459	(TTA)5	F:ACGTGTCGATTTCAATTCAACCA	60	/	/
		R:AAATTGCAAGTGTGGTGGGATC			
*BG0464	(GA)9	F:AAATATCACGGTCGGTTGTCCA	60	1	125
		R:TATTCGAGGGCTCCCATGAATG			
*BG0466	(GA)13	F:ACTACACGTACGTAATTTTGTGTGT	60	1	260
		R:TCGTTGTCCCAATTGATAGCCA			
*BG0473	(AG)6	F:AGAATCAGCGATTCCGTGAAGT	60	1	149
		R:GTTCAAAAACGACCTCTGTCCC			

*BG0477	(TC)6	F:TCTTCCACTGTCTCCTCGATCT	60	1	267
		R:CAGAGAGGCACATACGAGTGAG			
○BG0480	(AT)7	F:ACAAAATAAGAGTGGTTGGCCA	60	/	/
		R:AAAAACCAAATAGCGGGTGGC			
○BG0481	(TA)9	F:TTGAAGGATGATTTTTACTGTCACA	60	/	/
		R:TGCCTAGCGCCAAAAATGTTTT			
*BG0482	(ATT)7	F:ATTAGAGACCGGCTTGATGCTT	60	1	261
		R:ACGTGATATCTGTGGGCGTAAA			
*BG0486	(TA)6	F:CGTTTGAAATCTGCCCCACTTT	60	1	259
		R:CCAAAGAGTGTCAAAGTTGCCA			
○BG0489	(CT)6	F:CCTTGCTTGATGTGCCTTTGAA	60	/	/
		R:TGGCGGATTTTGTTTGAAGGTC			
○BG0493	(TC)8	F:TTCCCTCCCTGAATCCTCTTCA	60	/	/
		R:ATTGGAGAGATCTTCGGGGAGA			
*BG0495	(AT)11	F:CGCATGGCAAACTATTTCCGAA	60	1	259
		R:ACCCCTACTTCACTTTCATGGT			
*BG0501	(GA)7	F:TCAAATGTACAGGCTCATCGAT	60	1	278
		R:TTGCCTTGAACTGCGTGAAAAT			
○BG0504	(TA)7	F:GTAGACTTGTAGGATGGGGTGG	60	/	/
		R:GTTCATCTATAGCAAGCACGCG			
○BG0506	(TTA)5	F:GGAGAAGAATCAAGAGCACCCT	60	/	/
		R:CCCCCAACTCCCCAACTAAATT			
○BG0507	(GCA)7	F:AGAAACTGGGACTTGCGGTATT	60	/	/
		R:ATTGAGTTCCCAAGCACCTGTT			
◇BG0515	(TC)6	F:AGGAGCAATTCACTGTAATTAACAA	60	2	170-182

		R:GCTACACTTCGACAAATATGCA			
*BG0520	(ATA)6	F:TCTCAAGAGGACTTCATGTGAAT	60	1	191
		R:CTTGGAAAAAGCCCCGAGAATG			
*BG0523	(AG)7	F:CCGAAAACCCTCCAATGCTTAA	60	1	126
		R:ATCTCCCTCTCTCAGACAGGAG			
*BG0527	(AT)6	F:GTTCGCGAGCCTAGTAGGTTAA	60	1	249
		R:ACACACACCTTGATACATACATTCT			
○BG0528	(GA)7	F:AGCGCCAAAGAAATCAAACGAA	60	/	/
		R:CGATCTCCCTCTCTTTCTCACG			
○BG0529	(CT)9	F:CGAGTAGACTACGTGTAAGGCC	60	/	/
		R:GAGAAATGTATCAGTGGTGCCG			
◇BG0530	(AC)6	F:GGTGGAGGAGATGTACGACAAA	60	2	247-269
		R:CTGTCCCGATCGATTGTGTTTG			
○BG0532	(AG)7	F:GGAAGAGGTACTGCGGTGTATT	60	/	/
		R:TTCTAACGCTTACGCTCTCGC			
*BG0534	(GAA)5	F:ATCGGGTATAATCCGGAAAGGC	60	1	164
		R:CCCACTGTCGTCCCTTGTTATT			
*BG0537	(AT)11	F:TGTTAGTTTGAGAAGGCAAAGTGT	60	1	113
		R:AAGCAAGTACCTTACCTGGCAA			
○BG0539	(AT)10	F:GGGGTATATGTGATTAGCGGGG	60	/	/
		R:TGAATTAAGTGTCTCAGCCCCC			
*BG0541	(CT)9	F:CCCAAAACCCTCCTTCCTCTC	60	1	204
		R:AATCACGCATCTCTCTCGTCTC			
*BG0543	(CT)6	F:ACGCTTATACAGACGCATAGCA	60	1	169
		R:TGAGAACAGAGGAAGACACAGC			

*BG0557	(CT)15	F:AAACCCGCTGCTTGTACAGATA	60	1	175
		R:TGCAATTTAAATGCAAGCCCCA			
○BG0558	(TC)9	F:CACGTCAGCACCACACCTATAT	60	/	/
		R:GGGAAGGGAGGGAGAGAGATAA			
*BG0560	(TA)6	F:TGCCAATTTGAAGACCCTCGTA	60	1	189
		R:TTGACTTCAGTTGTGCCCCATA			
○BG0565	(AG)6	F:GGATTCAGGCACAGAAACACAC	60	/	/
		R:GTTATCGTCATGGTTTCGCTGG			
○BG0567	(ATC)6	F:ACTACGAGTAGACTCTTCTTTGCT	60	/	/
		R:GCTGTTGGTTCTCTCAAGCCTA			
○BG0568	(AT)7	F:ACATAAACATTACATTTGGGAAGCT	60	/	/
		R:GATAGTTTCGAACCACCCCCAA			
○BG0570	(TA)7	F:GGTAAAGATGAGAGGCGAGAGG	60	/	/
		R:CACCCCATACCCCCTTTTTGTA			
*BG0571	(TTA)5	F:CCAAATCCCCACAGTCGACATA	60	1	116
		R:TCGAATTTGGTGTCGACAATGA			
○BG0576	(AT)6	F:AGAAAGCCCCCAGTAATGAGTG	60	/	/
		R:CCCCCTCCCCCTAATTAGGTAT			
○BG0578	(AT)6	F:CTCACGTACATACCTTGCCACT	60	/	/
		R:AATGGAGTTGAACTATGACCGT			
◇BG0579	(AAG)5	F:ACGGGACTAAACACACCGATTT	60	4	218-230
		R:GCATCATCCATGGCTGTTCATC			
*BG0580	(CT)6	F:GGTTGTTCTTGCAACACTCTCC	60	1	102
		R:CAGGGGCAATGGCATTAACAAA			
*BG0582	(TA)9	F:AGAGCTAATCCCGTGTGAACAG	60	1	244

		R:TCCTCCCCTATGATGACTCGTT			
*BG0584	(TG)6	F:ACTACGAGTAGACTTATTGAACACA	60	1	146
		R:TCATGATTCCTCTGATTGGCATCA			
*BG0586	(TC)12	F:AACAAGTTCGTCGTCGGTGATA	60	1	214
		R:CTGCAGGAGTCGAAGGATTCTT			
*BG0587	(AC)7	F:AAGTTTGGGGGTATGCTACGAG	60	1	245
		R:ACATTCTAAGCTCGCCCAATGA			
○BG0593	(AC)6	F:AGTTTGGGGGTATGCTATGAGC	60	/	/
		R:AGTTCCCAGATCAACCCCAAAT			
○BG0595	(AT)13	F:AACGCTTTAAGCAGTTGCAGTT	60	/	/
		R:ACATTGTGATAGAGGGAGCCTT			
*BG0601	(AGA)5	F:ACGAATTTATGGAGAAGGCCGA	60	1	261
		R:CCAGGATCTTCACTGGACGTAC			
*BG0604	(TA)6	F:TTGACTATTAGTGTGGCGACCC	60	1	200
		R:CAGCTCCCAGTGATGTTTCTCT			
○BG0605	(AT)14	F:GGACCATTCCAAAACACATGCA	60	/	/
		R:TCTGTGCTATTAGGTGTGTCTTT			
*BG0607	(AT)7	F:CGCACGACCAGAAAATAGGAAC	60	1	261
		R:AAGCAGGTACACCATTCCTCTG			
*BG0608	(TA)10	F:GGAGCCCTTTCACAATTGCTTT	60	1	123
		R:TGTGATGTATTACATTGTTTGAGCA			
*BG0612	(TTA)5	F:AGCAATTCAGGCGTGAGAGTAT	60	1	274
		R:TTTACACGATCCGAGAATCCGA			
*BG0613	(AT)7	F:CGTTTGCAATGTCAACCCTCTC	60	1	244
		R:GTAAATGAGACATGCAGGCAGC			

*BG0622	(CT)8	F:TGTTCACCATTGATGTGGAAGTC<br>R:AGTCTAGTCCAAGGAAAGGAGAGA	60	1	233
○BG0624	(AT)8	F:TTTTTGAAGCATGCCCACTTGC<br>R:GGAGAGATGACACAGCTGGAAT	60	/	/
*BG0629	(TAT)5	F:GCAAGAGTATGGACAGGAGGAG<br>R:AAGACAATTCCCAGGCGTCTAG	60	1	202
*BG0635	(ATA)5	F:GCCAAAAACACTGCAGGTAACA<br>R:TTAGGACACCCCTTCTGAGACA	60	1	224
○BG0636	(CA)9	F:ACTGCATCCCTCTAAAAGTGAGA<br>R:AAGGAGGAAGAGTTGTTGGTGG	60	/	/
◇BG0641	(TCA)5	F:TTTGGACTTTCTATGCCGTCCT<br>R:AGACTGGTTTTCATGACACGGA	60	2	248-254
*BG0644	(GA)11	F:AGGCATTCTTCTCGGATAACCA<br>R:CTGCCCACTAACAATGAATGCC	60	1	207
○BG0652	(AAT)6	F:AGTGTGTACAACTGGTTACGGT<br>R:AGCTCACGCAGACTAATGAGAA	60	/	/
*BG0653	(TA)18	F:ACTATGCATGGCTAGCTTGTGT<br>R:ATGCATAACAACACAAGCAGGG	60	1	248
*BG0656	(AC)6	F:AAGTTTGGGGGTATGCTACGAG<br>R:AATCACCAGTTTCCCAGATCCC	60	1	224
○BG0662	(AT)6	F:TCAGTTGAAGACCGAGTAAGGC<br>R:ACTTCATCTTCATCTGTTCATTTCA	60	/	/
○BG0663	(TAT)5	F:AATCCCGAGTCTCAACCAAACA<br>R:TGTGTTTGATGTCGTGGGTTTG	60	/	/
*BG0664	(TC)6	F:TGCAAATTCCGCACCTCATTTT	60	1	170

		R:GAAGTGGCGCCAAGATATCAAC			
*BG0668	(AT)11	F:ACGAGTAGACTAAATTAGGGGTCT	60	1	245
		R:AGTTGCTCGAGACTTGTGATGT			
*BG0669	(TA)6	F:TCCTAATTCACCATCTGATTAGCT	60	1	241
		R:CCATGTGGCAAGCTTCAATGAA			
*BG0670	(TTTC)5	F:ATTTTGGCAGTGCGCTGTAATT	60	1	160
		R:GATGGCCTTGTGAATGCAAACT			
*BG0672	(TA)6	F:AGACTACAACATAAATCAGGCACT	60	1	222
		R:TCTCAGGTGCTATCTGACCAGA			
*BG0677	(GTT)6	F:TGCAAGAGGTGGGATGTAAAGT	60	1	123
		R:TGTTATGGCCCCTTTTCTGGTT			
*BG0678	(AC)6	F:CCGCAAATTCCCCCAAAGAAAA	60	1	223
		R:CGAGTTCTGAAGGGAGTTTTGC			
○BG0679	(ATC)6	F:ACGATCTATTCTACCTCTATCAGCA	60	/	/
		R:AACACTACGGATACAAGGCTGG			
*BG0686	(AG)7	F:TGCCCAAACAAAAAGACAAAAAGA	60	1	220
		R:AAAGAGCCGGATGTAGGTGATT			
*BG0689	(AT)6	F:TCTCTTTTTGGTTCATGTTAGCCT	60	1	216
		R:GCGCAAACGTCTTCCATTTTCA			
◇BG0704	(GA)6	F:CGTTTCCAAGAAGATCAACGCA	60	2	253-257
		R:TGGTGATGATGACTTTTTGGGC			
*BG0708	(TA)7	F:GCCCAGATTTTATCATTGCCGG	60	1	275
		R:ATGGACCCCCTGATCTTGAAAC			
○BG0709	(AT)7	F:ACAAGAGGAAATGATGGGTTTGT	60	/	/
		R:TTAGTCCCCCTCCTGAGAATGT			

*BG0710	(CA)6	F:TCATGAGAAGACCATTCCACCG	60	1	122
		R:ACGCATAGTTCCTGCCCATTAA			
*BG0712	(TA)10	F:CTACTCCCTTCGAGGATCAGGA	60	1	166
		R:AGGATTTGCTTGACAGGGGTAG			
*BG0715	(TC)9	F:GGAGGGGGTCAAAATGTTCTCT	60	1	259
		R:CCCACATGAGCACCTATTGAGA			
*BG0716	(AT)6	F:TAAAGGCCTAAGAGCTAGCTCG	60	1	266
		R:ACCTTAATTTTGGCTAAAGACGGA			
*BG0718	(AAT)5	F:ATAGCCTGTCCCATGCTTTCAA	60	1	210
		R:CCAAGCCGAGTCTAACATCAGT			
*BG0720	(TA)8	F:CTCGGTAAACCACTTCCCTCAA	60	1	236
		R:TGGAAGGTAGTAAGAATTTGGGAGA			
○BG0722	(TC)6	F:CTGTAGCTAGTGATCTGGGTGG	60	/	/
		R:CGAGAGAGATGAGAGCTCTTCG			
*BG0724	(AT)8	F:TTAGGCAGGGTCCTTATGTTGC	60	1	157
		R:TTTAAATCGGACCAAATGGCCC			
○BG0725	(TA)9	F:GGGTCACGTCTTTCCTCTTTCT	60	/	/
		R:GAAAAACCCAACACGTACAGCA			
*BG0727	(TA)6	F:AAATCGCAAACTCAAACGGACC	60	1	221
		R:GAGTGGCATATGTGAGAGCAGA			
*BG0731	(TAT)5	F:AGTTAAATTTTCAGCTCATTCGGT	60	1	277
		R:TGTGGAGATAAATATTGGTTGGTGG			
◇BG0734	(AT)12	F:GAGATATAAATGTGGGCATGGCA	60	4	248-256
		R:ATGCCTCACAACTTAGTAGGGC			
*BG0735	(ATT)5	F:CGATAAGAATGGTGCAAGCCAG	60	1	223

		R:AGGGATAGAAAACCACTCATAGAGT			
*BG0740	(TA)9	F:ACATGTGGCAGACTAGCATGAA	60	1	200
		R:TCAACAAATTAGAGGACACTGCC			
○BG0741	(AT)6	F:ACAAGCAACATGACGCAAGAAA	60	/	/
		R:CCTACCCCAGTTTCCAATGTGA			
○BG0744	(AG)6	F:TCTATATAGAGGTCGAGCGCGA	60	/	/
		R:TTCATACCCATGGACCCAAGTG			
○BG0746	(TA)6	F:TTAGGAGTGGCAAAAGACCCTC	60	/	/
		R:TCTTGTCTTCTGCTTCGACGAA			
*BG0747	(TAT)5	F:TCCATTTCGGTTCTACATGCCA	60	1	185
		R:TGCATGCCACATCTTTTCAATCA			
*BG0752	(AAT)9	F:AAATGGTACCGAGTTTTTGGCG	60	1	127
		R:CTCTCACGCCAAGGTGAATAGT			
*BG0753	(TC)12	F:GAGTAATGCGACGGACAACATG	60	1	151
		R:TACATTCAACGGCGGTAGTGG			
*BG0754	(TA)6	F:GTCATGTGCCTTACAGTTGCAA	60	1	280
		R:GGTACCAACGGACATTGCAAAA			
◇BG0755	(AT)6	F:TCCCTTGTAGCCCTCTCTCTTA	60	2	271-273
		R:TTGTGGTCGGTTTTGCAAAACT			
○BG0762	(ATT)6	F:ACGTGTATTGGGTTAAGTCGCT	60	/	/
		R:ACCCTCACATTTTATGCTTCGT			
*BG0764	(ATT)6	F:CGAATTTCATGATCTCTCGGCA	60	1	266
		R:ACGTTTCAGTACGAGGCTCTTT			
*BG0769	(AATA)5	F:AAGGTGGGTCTTTCATGCTCTT	60	1	115
		R:TGGAGTTAGCAAAATTGGCCAC			

*BG0770	(TA)7	F:TCCCGATGAATGTTTCAGTGGA	60	1	222
		R:ACCGTCAAGAGTCCAAAACTGT			
*BG0772	(AT)6	F:AGGGTTGATTGAGACTGTCACT	60	1	222
		R:TCAATTTGCAAACGGCTCTTGT			
*BG0778	(AG)16	F:GACTCATACGACCCAACCTTGT	60	1	155
		R:TTGGTTTGTGGGCAATGGTTAC			
*BG0788	(AAT)5	F:CAAATTAGCGAACGGTAGCTCG	60	1	127
		R:AGCCGTCTAGTCAACAGATAGC			
○BG0789	(AAT)6	F:TGGCCAATTGGGTGGTAATTTT	60	/	/
		R:AGTCCAAATACCCTCAACCACC			
○BG0791	(TA)10	F:TACTAACCTCAAGCACATGGCC	60	/	/
		R:CATCCTGCATGTCAAGCTCAAA			
*BG0792	(AT)13	F:AGTAGACTGGGTTCCTCGTCAT	60	1	221
		R:AGGCTAATGCTCTTGCCTTTCT			
○BG0794	(TA)6	F:TTTTTATTGGGGGTGGCTCTGT	60	/	/
		R:ACACCTACACATGGCTTTGTCT			
○BG0796	(GA)8	F:CCTCAAAAGTACAGCGAGGACT	60	/	/
		R:TTCGGACTTTTTGTAAGCGCAG			
*BG0800	(AT)6	F:GTGTGGCTATTCGGAAACAACA	60	1	278
		R:CGTTCCCTTCTCATTGTTCCTC			
*BG0804	(GAA)7	F:CGTGAGTAACGCTCTTTCACTC	60	1	263
		R:AATGTTGAGAAACGTTGGCGAG			
○BG0807	(GT)7	F:GTTCAACATTCTAAGCTCGCCA	60	/	/
		R:GGGGGTATGATACGAGCCATAC			
○BG0808	(TA)6	F:GGAGAGATGGGTTGATGTGTGT	60	/	/

		R:GAAGCTCATATCACCCCAGTCC			
*BG0809	(TTC)5	F:TGGTTGGGAAGTGCAATTTTCC	60	1	223
		R:CCACTTTGCCCCAAGTCAATTT			
○BG0811	(TC)8	F:CTCGAACTTGCGTCTCTCTTCT	60	/	/
		R:ATTCAAGTGACAAGATGCGTGG			
*BG0812	(AT)8	F:ACAAAGTAGACATGGAAGGCGA	60	1	252
		R:AAAATTGCTCGTCTAGGGTGGT			
*BG0814	(ATT)6	F:TTTGAAATCACTGAACCACCGC	60	1	258
		R:GTGTGCCCCCTTGTTCTACTG			
*BG0815	(TC)6	F:GACTCTAATTTCCTGGGGCTCC	60	1	240
		R:TATGCACATGAGTCTAGCCCAC			
○BG0820	(ATT)6	F:GTGTACAGTCGAGTTCGTTTGC	60	/	/
		R:AGTGAAGAAGATGGGTGTGTTT			
*BG0822	(AC)8	F:TCCCCCTGGTATGATCTTTCCA	60	1	221
		R:GTTCAACATTTAAGCTCGCCCA			
○BG0823	(TA)11	F:CGAGTAGACTGAGAACGAAGACA	60	/	/
		R:ATTGTACTTCACGTCAGGCCAT			
*BG0825	(ATT)5	F:AATAACATGTTGCTGTCACGCC	60	1	132
		R:TCACTTCTCCTCCAAGAAAAGAA			
*BG0827	(TA)6	F:ACTACGAGTAGACTGAATACTCACA	60	1	253
		R:AAAACAGTGTCAGCCCCAGTAA			
*BG0829	(AT)11	F:TGAACAGTACTGCTCAGCAACA	60	1	213
		R:AGGAGAGAGAAAGGCTAGGTGT			
*BG0832	(AT)7	F:ATTCGTGGGTATTGTCGAGTCC	60	1	150
		R:CATCATTTGACTCAATCCGGGC			

*BG0836	(TCT)5	F:TAGGAAAGGGTAGTGCTTGCAG	60	1	176
		R:TGCACATTATACCCATATTGAACAA			
*BG0837	(TA)6	F:CGTAATCTCAAACGCACCAGTC	60	1	223
		R:GGCAATGCCTATCAATCCTTACA			
*BG0838	(GA)8	F:CGAGTAGACTGATACTGCCGTT	60	1	226
		R:TCCTCTCCTTCTCTCACAGTCC			
○BG0839	(TA)11	F:TCCGAAAAAGACAATGAATGCCT	60	/	/
		R:TCCACTACCGAGTTTGTTGACC			
*BG0840	(CCT)6	F:CTGCCCATCAAAATGCAAGACA	60	1	273
		R:AGTCGAGAAATCTGTTGTGCCA			
*BG0843	(AT)9	F:ACTCTTGGCAAATGACCTCAAG	60	1	233
		R:CTTTGAAAGACTCTTCCAATTCAGT			
○BG0850	(AT)9	F:ACTCTCTTTGGTCTTGTTGTCCA	60	/	/
		R:CTTCCAAGTTTGATGAGTGCCG			
*BG0851	(CTTT)5	F:CCTGGGCAGCAACACATTTAAT	60	1	226
		R:TGTGCTACATATGCTGTGATGT			
*BG0853	(GA)6	F:CACATGCAGTGTAATTGACGCA	60	1	182
		R:GGTGAGAGTGAGGGAACATGAG			
*BG0858	(GA)8	F:GAGTCGAGAGATAGCTTCAGGC	60	1	221
		R:TCAATGTTTCACGGTCTCACCC			
○BG0859	(TA)6	F:AACCTCATCCATCGCTTTCAGT	60	/	/
		R:TTCTTTGGAAGACTACTGGGCC			
*BG0860	(TC)6	F:TGTTTTCGTCTTTCTGTTGGCC	60	1	233
		R:CCGTGGTTTTTGACAGAAGTGT			
*BG0861	(AAT)5	F:CACAGTGGAGAGAGAGGAGAGA	60	1	215

		R:CCCCAACCCAGTTTAGCTAGTT			
○BG0862	(TC)9	F:ACGAGTAGACTTCTGCTTCAATGA	60	/	/
		R:AGCCAATGATCTGCAGCTTTTG			
○BG0865	(AG)6	F:TTGCTGGATCTCCTTGGTTCAA	60	/	/
		R:AAATTTGCAGAAGAACAGCCCC			
*BG0869	(TA)6	F:TGCACTTCTTCCCTTTTCAGTT	60	1	106
		R:TTTCGCTTTCCCTATTCTCCCC			
*BG0870	(ATT)7	F:TCTATCAGGAACATGGCCCATG	60	1	211
		R:TGTCACAATGCCCAAGTTGAAC			
*BG0875	(TG)6	F:GGCATGTTTCAGAATGGCCAAT	60	1	123
		R:AGATCTCAAGTTGGCCCTCTTG			
○BG0886	(TA)8	F:TCAGTGCATGCATGTATGTGTG	60	/	/
		R:CATGAGTCAGTCCAGTTCCACA			
○BG0892	(CT)7	F:ATTCCTTCAAACAACGAAGCCG	60	/	/
		R:GTCTTAAGAGAGAAAACGAGGTGT			
*BG0894	(TC)6	F:GGTGGCAAATTGTGAGGTTAGA	60	1	212
		R:TAGGAGGAAGAGAAGGATGGGG			
○BG0896	(AT)18	F:GCTAACGCACGAAAGGATTTGT	60	/	/
		R:AGCTTTGAATTCACTTGGAGGT			
*BG0900	(TAT)5	F:CAGTGGATCCCTCCTACCTAGT	60	1	152
		R:GAAGGTGCAGATTTTCAACCCA			

Note: *, the primers generating monomorphic markers in 24 samples; ○, the primers with weak fluorescence signals in amplified products of most samples; and ◇, fixed heterozygosity.

Table S3. Validation of 19 SSR markers developed previously in 24 samples of *Betula alnoides*.

Primer Name	Motif	Primer Sequence	Ta (℃)	N_A	H_O	H_E	*I*	Size of allels (bp)
BAG01	(TG)8(GA)13	F: CAAGGTGCTCAGGGTGTTT<br>R: TATCCTCTTTGGCATTGAA	52	7	0.79	0.62	0.80	241-255
BAG02	(TC)15(CA)9	F: CCTTGCTCTGCGTGATGTATTT<br>R: CAGAATCCTCTTATTTCCACAG	52	8	1.00	0.81	1.83	228-244
BAG03	(CT)17	F: TGCGTATGCTTCCAACAC<br>R: ACCCAGAACCTGTCCACC	52	9	0.91	0.79	1.96	322-344
BAG04	(TC)21	F: TCTTTGCCTTAATGATTGTTGC<br>R: GGCTGACGGACACTGTTTGA	52	14	1.00	0.92	2.12	209-235
BAG06	(CG)5(CA)8	F: TCGCACATACACTCACACATT<br>R: ATGCTGCACACTTTTGATTTA	50	4	0.96	0.76	0.78	168-174
BAG09	(AT)5 G(TG)8	F: TCGTTCAGGCAACTCTATCAAT<br>R: ATTTGTAAAGATGGATGGAGAA	54	8	0.65	0.55	0.84	273-297
BAG10	(AG)5 … (GA)25 … (GA)	F: ACCAACTCCTAAACCACCATACC<br>R: GGGAGGATTTCAACGGCATTTA	54	16	0.74	0.88	1.97	215-263
BAG12	(TCG)5	F: GTCCTCCTTCACTATTCCTTTGT<br>R: CTTTCAGGGTAATTCTGATTTGG	52	5	0.79	0.66	0.76	163-178
BAG14	(TC)7(CT)9	F: CATTGCTTCATTTTGGCTTCTA<br>R: TTCTGATGTGAAATACTGCTGG	50	10	1.00	0.92	0.84	250-270
BAG16	(TG)8(AG)8 T(AG)6	F: CACTAACCGCAACCAATAATCC<br>R: ATTTGCCTTCCTACCAACTCTG	54	9	0.91	0.90	1.02	293-309
BAG17	(AG)15	F: GTTGGTAGTTGTGTGATGGA<br>R: AGTTGTATGGATAGAAAAGT	52	9	1.00	0.94	1.30	201-217
BAG18	(GGT)5	F: GGATGGGTGACTAAGAGGGAGGA<br>R: GCAATGGTAGACAAAGTCACAGC	50	3	0.71	0.56	0.77	248-260

BAG20	(TG)9 TT(AG)13	F: TTCTCCCACAACCCTTGATGC	50	11	1.00	0.84	0.98	249-273
		R: CCAGGCATCCAGTCCCTTATTA						
BAG22	(TC)15	F: CGACGACGATTACAACGAGT	52	11	0.88	0.77	0.95	247-275
		R: TGGGTTGACATTCTAAGGGTGCT						
BAG24	(TC)21	F: GAATTATTCGTGTTCAAAGTCT	52	9	1.00	0.76	1.12	242-258
		R: CATACAAGCAGTCAACCCATCT						
BAG25	(CT)5	F: GATCTCGCCGTCGTCTCAAC	52	5	0.50	0.62	0.86	243-265
		R: CCAACAGGCAACAGATACAGAGT						
BAG27	(TC)10 A(CA)10	F: ATGTGGAAGAGAGGATAGAATCT	50	4	0.91	0.77	0.69	317-325
		R: TGGAGTTAAGAGAACACAATAGA						
BAG28	(AC)7	F: ACCTATTACATTGAATTCTATGA	52	8	0.75	0.79	0.93	302-316
		R: ATCATCTGTTAGCTTATCATAAT						
BAG29	(AC)10	F: AGGCTTTGGCTTCCCTTATT	52	5	0.67	0.81	1.04	143-157
		R: CGGACACTTGTTCCGATTTT						

Table S4. Amplification rate of 96 SSR markers for *Betula alnoides* in six related species of genus *Betula*.

**Primer Name**	**Species**					
	Betula platyphylla	***Betula austro-sinensis***	***Betula cylindrostachya***	***Betula fujianensis***	***Betula hainanensis***	***Betula luminifera***
BG0009	+	+	+	+	+	+
BG0029	+	+	+	+	+	+
BG0032	+	+	+	+	+	+
BG0048	−	+	+	+	+	+
BG0070	−	+	+	+	+	+
BG0086	+	+	+	+	+	+
BG0091	+	+	+	+	+	+
BG0096	−	+	+	+	+	+
BG0106	+	+	+	+	+	+
BG0118	+	+	+	+	+	+
BG0119	−	+	+	+	+	+
BG0121	−	+	+	−	−	+
BG0128	+	+	+	+	+	+
BG0129	−	+	+	+	+	+
BG0148	+	−	+	+	+	+
BG0163	−	−	−	−	−	−
BG0165	−	+	+	+	+	+
BG0176	+	+	+	+	+	+
BG0188	+	+	+	+	+	+
BG0189	−	−		−	−	−
BG0205	−	−	−	−	−	−
BG0207	−	+	+	+	+	+

BG0222	+	+	+	+	+	+
BG0236	+	+	+	+	+	+
BG0238	−	−	−	−	−	−
BG0248	+	+	+	−	+	+
BG0252	+	+	+	+	+	+
BG0260	−	−	−	−	−	−
BG0267	−	+	+	+	+	+
BG0295	−	+	+	+	+	+
BG0296	+	+	+	+	+	+
BG0299	−	−	−	−	−	−
BG0314	−	−	+	+	+	+
BG0324	+	+	+	+	+	+
BG0325	−	+	+	+	+	+
BG0328	+	+	+	+	+	+
BG0329	−	−	+	−	−	+
BG0350	+	+	+	+	+	+
BG0354	+	+	+	+	+	+
BG0362	+	+	+	−	−	+
BG0367	+	+	+	+	+	+
BG0377	+	+	+	+	+	+
BG0386	+	+	+	−	+	+
BG0392	−	+	−	−	−	−
BG0395	−	−	−	−	−	−
BG0397	+	+	+	+	+	+
BG0400	+	+	+	+	+	+
BG0407	−	+	−	+	+	+
BG0412	−	−	+	+	+	+

BG0415	+	+	+	+	+	+
BG0416	+	+	+	+	+	+
BG0419	+	+	+	+	+	+
BG0422	−	+	+	+	+	+
BG0427	−	−	+	+	+	+
BG0432	+	−	−	−	−	+
BG0434	−	−	+	+	+	+
BG0437	−	−	+	−	−	+
BG0456	+	+	+	+	+	+
BG0461	+	+	+	+	+	+
BG0463	−	+	+	+	+	+
BG0470	+	+	+	+	+	+
BG0476	+	+	+	+	+	+
BG0478	+	+	+	+	+	+
BG0479	−	+	+	+	+	+
BG0485	−	+	+	+	+	+
BG0497	+	+	+	+	+	+
BG0505	+	+	+	+	+	+
BG0509	−	−	−	−	−	−
BG0514	+	+	+	+	+	+
BG0524	+	+	+	+	+	+
BG0525	+	+	+	+	+	+
BG0533	−	−	+	+	+	−
BG0536	−	−	−	+	+	−
BG0540	−	+	+	+	+	+
BG0544	−	+	+	+	+	+
BG0545	−	−	+	−	−	+

BG0546	+	+	+	+	+	+
BG0547	+	+	+	+	+	+
BG0548	−	−	−	−	−	−
BG0550	−	−	+	+	+	−
BG0551	−	+	+	−	−	+
BG0554	+	+	−	+	+	+
BG0559	−	+	+	+	+	+
BG0563	−	+	+	+	+	+
BG0566	+	+	+	+	+	+
BG0572	−	+	−	+	+	+
BG0573	−	+	+	+	+	+
BG0574	−	−	−	−	−	−
BG0577	+	+	+	+	+	+
BG0583	+	+	+	+	+	+
BG0589	−	−	−	+	+	+
BG0592	−	+	+	+	−	+
BG0598	−	+	+	+	+	+
BG0606	+	−	+	+	+	+
BG0888	−	+	+	+	+	+
BG0893	−	−	−	−	−	−

Note: +, present of PCR amplicons; −, absent of PCR amplicons.
